# Evaluation of common protein biomarkers involved in the pathogenesis of respiratory diseases with proteomic methods: A systematic review

**DOI:** 10.1002/iid3.1090

**Published:** 2023-11-20

**Authors:** Hadi Rezaeeyan, Masoud Arabfard, Hamid R. Rasouli, Alireza Shahriary, B. Fatemeh Nobakht M. Gh

**Affiliations:** ^1^ Chemical Injuries Research Center, Systems Biology and Poisonings Institute Baqiyatallah University of Medical Sciences Tehran Iran; ^2^ Blood Transfusion Research Center, High Institute for Research and Education in Transfusion Medicine Iranian Blood Transfusion Organization (IBTO) Tehran Iran; ^3^ Trauma Research Center Baqiyatallah University of Medical Sciences Tehran Iran

**Keywords:** biomarker, pathogenesis, proteomics, pulmonary disease

## Abstract

**Aim:**

Respiratory disease (RD) is one of the most common diseases characterized by lung dysfunction. Many diagnostic mechanisms have been used to identify the pathogenic agents of responsible for RD. Among these, proteomics emerges as a valuable diagnostic method for pinpointing the specific proteins involved in RD pathogenesis. Therefore, in this study, for the first time, we examined the protein markers involved in the pathogenesis of chronic obstructive pulmonary disease (COPD), idiopathic pulmonary fibrosis (IPF), asthma, bronchiolitis obliterans (BO), and chemical warfare victims exposed to mustard gas, using the proteomics method as a systematic study.

**Materials and Methods:**

A systematic search was performed up to September 2023 on several databases, including PubMed, Scopus, ISI Web of Science, and Cochrane. In total, selected 4246 articles were for evaluation according to the criteria. Finally, 119 studies were selected for this systematic review.

**Results:**

A total of 13,806 proteins were identified, 6471 in COPD, 1603 in Asthma, 5638 in IPF, three in BO, and 91 in mustard gas exposed victims. Alterations in the expression of these proteins were observed in the respective diseases. After evaluation, the results showed that 31 proteins were found to be shared among all five diseases.

**Conclusion:**

Although these 31 proteins regulate different factors and molecular pathways in all five diseases, they ultimately lead to the regulation of inflammatory pathways. In other words, the expression of some proteins in COPD and mustard‐exposed patients increases inflammatory reactions, while in IPF, they cause lung fibrosis. Asthma, causes allergic reactions due to T‐cell differentiation toward Th2.

## Graphical abstract

Many diagnostic mechanisms have been used to identify the pathogenic agents of respiratory disease (RD). Proteomics is a diagnostic method to identify proteins involved in RD pathogenesis. In this study, for the first time, we examined the protein markers involved in the pathogenesis of chronic obstructive pulmonary disease, idiopathic pulmonary fibrosis, asthma, bronchiolitis obliterans, and chemical warfare victims exposed to mustard gas, using the proteomics method as a systematic study.

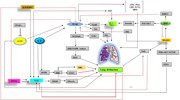

**Abbreviations**
2DE: gel electrophoresis
AHSG: α2‐HS‐Glycoprotein
ALB: albumin
APO C3: apolipoproteinC3
APOA1: apolipoprotein A‐1
BALF: bronchoalveolar lavage fluid
BCL‐2: B‐cell lymphoma 2
BO: bronchiolitis obliterans
COPD: chronic obstructive pulmonary disease
CXCL: C‐X‐C motif chemokine ligand
ELISA: enzyme‐linked immunosorbent assay
EMT: epithelial‐mesenchymal transition
ERK: extracellular signal‐regulated kinase
FTH1: heavy chain ferritin
GAPDH: glyceraldehyde 3‐phosphate dehydrogenase
GC: vitamin D binding protein
GSTP1: glutathione S‐transferase Pi 1
HSPB1: heat shock protein beta‐1
IGKC: immunoglobulin kappa constant
IL: interleukin
IPF: idiopathic pulmonary fibrosis
KRT9: keratin 9
KRT10: keratin 10
LC‐MS‐MS: liquid chromatography with tandem mass spectrometry
LCN1: lipocalin1
LXA4: lipoxin A4
MALDI‐TOF: MALDI coupled to time‐of‐flight
MAPK: mitogen‐activated protein kinase
miR: microRNA
mTOR: mammalian target of rapamycin
OSMR: OSM receptor
Pb: peripheral blood
PI3K: phosphoinositide 3‐kinase
PLC: phospholipase C
PPAR‐γ: peroxisome proliferator‐activated receptor gamma
PSMA2: proteasome 20S subunit alpha 2
RBP4: retinol binding protein 4
RD: respiratory disease
S100A8: protein S100 A8
S100A9: protein S100 A9
Src: short for sarcoma
TF: tissue factor
TF: transferrin
TGF‐β: transforming growth factor beta
UBE2N: ubiquitin conjugating enzyme E2 N

## 1 INTRODUCTION

Respiratory disease (RD) is one of the leading causes of patient mortality worldwide. RD includes many diseases such as those resulting from exposure to mustard gas, chronic obstructive pulmonary disease (COPD), Asthma, and many others.^1^, ^2^, ^3^ A wide range of clinical symptoms is observed in RD patients according to the type of disease and its pathogenesis. The pathogenesis of RD is not fully understood; however, several mechanisms and factors have been implicated in their development and progression.^1^, ^4^ Inflammation, coagulation, and oxidative stress pathways are known to play important roles in the development and progression of RDs. Studies have shown that some of these pathways, including inflammatory pathways and oxidative stress, are involved in developing COPD and Asthma. Activation of the NF‐κB pathway in COPD has been shown to lead to inflammation. Furthermore, a decrease in the expression of superoxide dismutase (SOD) in COPD has been found to lead to an increase in oxidative stress.^5^, ^6^, ^7^, ^8^, ^9^

Based on the evidence obtained, inflammation is the most important pathway in the pathogenesis of RD. Most cells in the respiratory pathway induce the production of cytokines. On the other hand, factors produced by respiratory cells lead to the production of inflammatory mediators that result in the chemotaxis of immune cells. Most oxidative stress pathways lead to inflammation. Despite disease progression, the occurrence of inflammation can also induce apoptosis.^10^, ^11^, ^12^

Pulmonary disorders have also been shown in patients exposed to mustard gas and idiopathic pulmonary fibrosis (IPF) due to the activation of inflammatory pathways.^13^ So far, common factors and pathways involved in the pathogenesis of RD have not been identified. It has also been found that RD, including COPD, has not been correctly diagnosed in many cases. Spirometry testing is not practical for diagnosing and differentiating COPD from other RD. Including warfare victims exposed to mustard gas, some patients have inflammation in the airways, similar to Asthma in terms of clinical symptoms. Therefore, it is difficult to distinguish between them.^14^, ^15^, ^16^ Therefore, there is an urgent need for efficient diagnostic biomarkers to differentiate between these illnesses and common indicators.

Various methods have been used to identify markers, but it has been shown that proteomics can help locate involved features in RD pathogenesis; it is a high‐throughput method for biomarker panel discovery with high sensitivity and specificity. On the other hand, it has been shown that identifying proteins in tissues and cells in various diseases can be used as a diagnostic method.^17^, ^18^, ^19^ Proteomics is an approach which dramatically has been used in recent decades to detect proteins in many diseases. Also, new proteins identified by this method can be effective in early diagnosis, follow‐up, and patient management. Identifying common protein markers in RD can be a suitable solution for designing target therapy and preventive strategies for RD patients.^18^

Furthermore, since inflammation is recognized as a primary pathogenesis pathway in RD, the identification of biomarkers associated with it can facilitate easier management of patients. On the other hand, the identification of factors associated with inflammatory pathways can be helpful in patient management.

No study has been performed to identify protein markers commonly involved in RD pathogenesis. Also, most studies have examined the protein markers involved in the pathogenesis of RD; the results of many of them are challenging.

Therefore, for the first time in this study, we examined the protein markers involved pathogenesis of COPD, IPF, Asthma, bronchiolitis obliterans (BO), and chemical warfare victims exposed to mustard gas using the proteomics method as a systematic study. Finding shared proteins among these respiratory disorders is the goal of this research, which will also help create common therapeutic approaches and a better understanding of disease mechanisms.

## 2 METHODS

### 2.1 Search strategy and literature

A systematic search was conducted up to September 2023, covering several databases, including PubMed, Scopus, ISI Web of Science, and Cochrane, from July to September 2023. Keywords used to search databases included the following: “COPD, Chronic Obstructive Pulmonary Disease, COAD, Chronic Obstructive Airway Disease, Chronic Airflow Obstruction, Chronic Obstructive Lung Disease, Obliterative bronchiolitis, Constrictive Bronchiolitis, Exudative Bronchiolitides, Proliferative Bronchiolitides, Mustard Gas, Mustard, Di‐2‐chloroethyl Sulfide, Sulfur Mustard, Yellow Cross Liquid, Yperite, Bis (beta‐chloroethyl) Sulfide, Mustard gas, Psoriazin, Mustard lung, Sulphur mustard, Asthma, IPF, Pulmonary Fibrosis, Fibrosing Alveolit, Idiopathic Pulmonary Fibrosis, Fibrocystic Pulmonary Dysplasia, Usual Interstitial Pneumonia, Proteomic, proteome, proteomic profiling, proteomic fingerprinting, proteomic biomarkers.”

Search strategies were created using the words AND and OR. It did not restrict publication time, language, and type of study. It exported the results of searching all databases to Endnote X7 software (Thomson Reuters). Then, by reviewing the title and abstract extracted, relevant articles. Screening of related articles using the titles and abstracts was reviewed by two authors (B. Fatemeh Nobakht M. Gh and Hamid R. Rasouli) and discussed cases of discrepancy with the third author (Masoud Arabfard). Also, to further investigate, it manually reviewed the references of selected articles.

The criteria considered for article selection were based on PICO (population, intervention, comparator, outcome, and setting) and included the following: population—patients with RD, intervention—proteomics, comparator—healthy individuals, and setting—case‐control study.

### 2.2 Inclusion criteria

Criteria used to select articles included: case‐control studies performed on individuals over 18 years, the use of proteomics methods to examine markers, and access to supplementary files.

### 2.3 Exclusion criteria

Exclusion criteria for deleting unrelated articles included: review studies, clinical trials, case reports, use of animal models for analysis, in vitro studies, studies without a healthy control group, studies on children, and lack of access to supplementary files.

### 2.4 Data collection

After selecting articles based on cited criteria, extract the following information: authors' names, publication year, sample size, country, age, sex, type of sample, type of technique used, statistical analysis, number of Up/Downregulation proteins, model characteristics, and validation.

## 3 RESULTS

**Figure 1:**
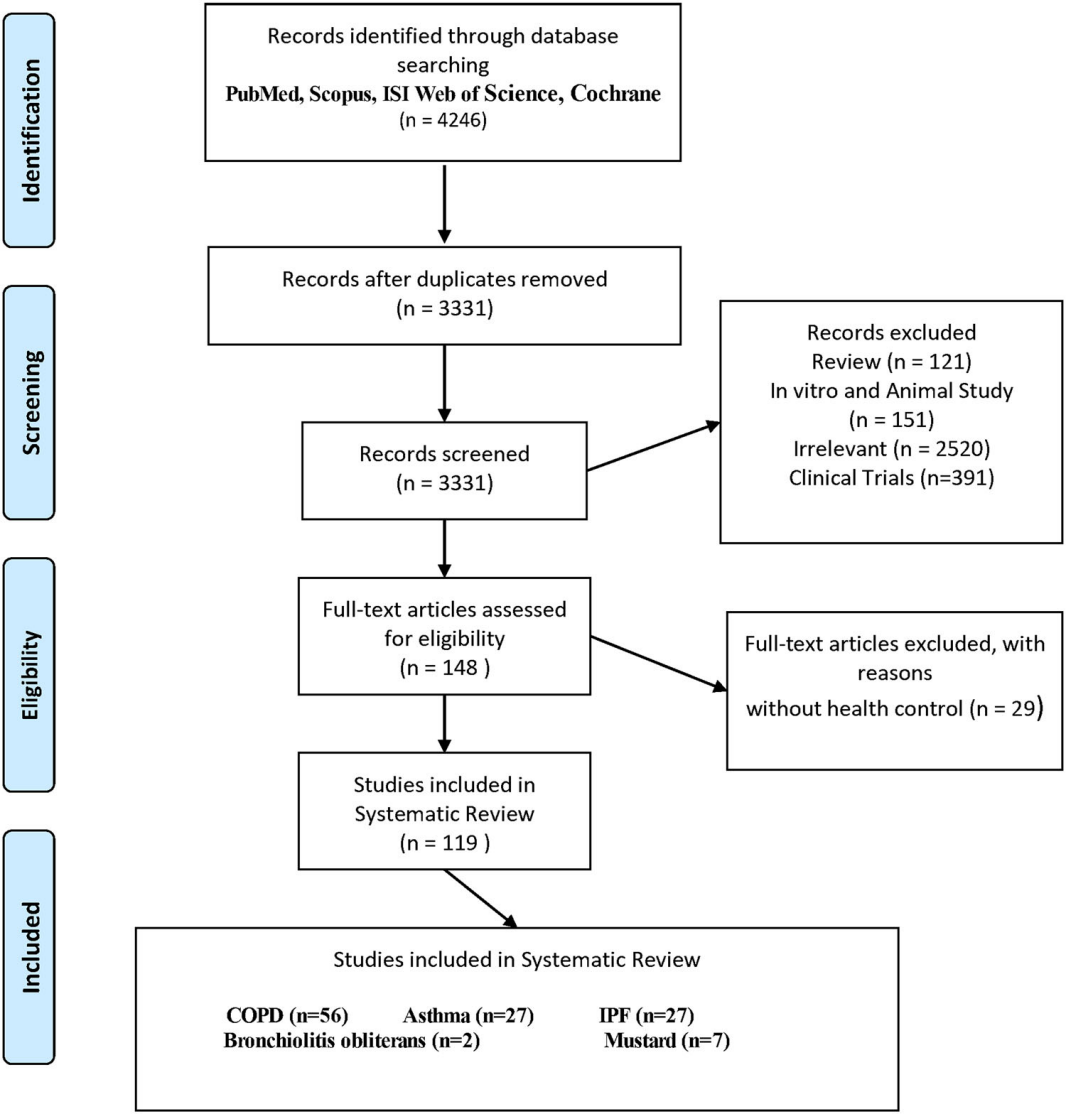
Prisma flow diagram.

This study examined five diseases of COPD, Asthma, IPF, BO, and mustard gas‐exposed victims. In total, 4946 articles entered endnote X7 software. After deleting the duplicates and other articles, reviewed the full text of 148 articles based on the reasons mentioned in Figure 1. Finally, 119 studies were selected in this systematic review. Details and related findings of selected papers (age, sex, sample type, type of technique used, statistical analysis, number of increased or decreased proteins, model characteristics, validation test) are shown in supplementary files.^20^, ^21^, ^22^, ^23^, ^24^, ^25^, ^26^, ^27^, ^28^, ^29^, ^30^, ^31^, ^32^, ^33^, ^34^, ^35^, ^36^, ^37^, ^38^, ^39^, ^40^, ^41^, ^42^, ^43^, ^44^, ^45^, ^46^, ^47^, ^48^, ^49^, ^50^, ^51^, ^52^, ^53^, ^54^, ^55^, ^56^, ^57^, ^58^, ^59^, ^60^, ^61^, ^62^, ^63^, ^64^, ^65^, ^66^, ^67^, ^68^, ^69^, ^70^, ^71^, ^72^, ^73^, ^74^, ^75^, ^76^, ^77^, ^78^, ^79^, ^80^, ^81^, ^82^, ^83^, ^84^, ^85^, ^86^, ^87^, ^88^, ^89^, ^90^, ^91^, ^92^, ^93^, ^94^, ^95^, ^96^, ^97^, ^98^, ^99^, ^100^, ^101^, ^102^, ^103^, ^104^, ^105^, ^106^, ^107^, ^108^, ^109^, ^110^, ^111^, ^112^, ^113^, ^114^, ^115^, ^116^, ^117^, ^118^, ^119^, ^120^, ^121^, ^122^, ^123^ It listed for COPD, IPF, Asthma, BO, and mustard gas‐exposed victims, respectively.

### 3.1 Biological specimens

Using different samples in 119 selected studies. Accordingly, in COPD, the most commonly used selection was peripheral blood (PB); serum, plasma, or mononuclear cells were also isolated (25 of 56 studies).^42^, ^43^, ^44^, ^45^, ^46^, ^47^, ^48^, ^49^, ^50^, ^51^, ^52^, ^53^, ^54^, ^55^, ^56^, ^57^, ^58^, ^59^, ^60^, ^61^, ^62^, ^63^, ^114^, ^119^, ^123^ Also used were saliva samples in one study. In IPF, the most commonly used selection was Broncho‐alveolar lavage fluid (BALF) (12 out of 27).^31^, ^32^, ^33^, ^34^, ^35^, ^36^, ^37^, ^38^, ^39^, ^40^, ^41^, ^45^ In Asthma, PB was the most commonly used sample (12 out of 27).^25^, ^26^, ^27^, ^28^, ^29^, ^30^, ^49^, ^50^, ^63^, ^115^, ^117^, ^124^ In one study, the exosome was used; in another, one nasal brush was used as a sample. The most commonly used selection for mustard gas‐exposed victims was PB (5 out of 7).^22^, ^23^, ^24^, ^58^, ^121^ BALF was used in one study, and tissue as a biological sample in another. BALF was used in BO in two studies.^20^, ^21^

### 3.2 Technological platform used

Proteomics is a technique that evaluates the proteins in the samples; they can be cells, solutions, tissues, and many different biological samples. Proteomics includes other techniques depending on the sample type and the study's purpose.^125^ In the studies included in the systematic review, various methods have been used. In COPD and IPF, the most commonly used techniques were Two‐dimensional gel electrophoresis (2DE) and liquid chromatography with tandem mass spectrometry (LC‐MS‐MS). In Asthma, LC‐MS‐MS was used more than any other technique (13 out of 27). Matrix‐assisted laser desorption/ionization (MALDI) coupled with time‐of‐flight (MALDI‐TOF) was more commonly used in BO (Supporting Information S1). In mustard gas‐exposed victims, four studies used 2DE, and 5 out of 7 studies used MALDI‐TOS‐MS/MS. Most studies used western blot and enzyme‐linked immunosorbent assay (ELISA) techniques for validation (Supporting Information S1).

### 3.3 Proteomic biomarkers

After selecting the articles investigated, the expression changes of proteins were identified by proteomics and, in COPD, altered the expression of 6471 proteins. Asthma changed the face of 1603 proteins. Also, in IPF, 5638 proteins, and BO and mustard gas exposed victims, their 3 and 91 proteins expression were altered, respectively. Alteration of protein expression, or increase in their face in five diseases according to the control group, was considered.

After evaluating the results, 31 proteins were common between five diseases. These proteins included transferrin (TF), glutathione S‐transferase pi 1 (GSTP1), ubiquitin conjugating enzyme E2 N (UBE2N), haptoglobin (HP), eukaryotic initiation factor 5A (EIF5A), keratin 9 (KRT9), apolipoproteinA4 (APO A4), albumin (ALB), apolipoproteinC1 (APO C1), angiogenin (ANG), protein S100 A8 (S100A8), fibrinogen alpha chain (FGA), heat shock protein beta‐1 (HSPB1), complement component 3 (C3), lipocalin1 (LCN1), C‐reactive protein (CRP), ficolin 2 (FCN2), adiponectin, C1Q and collagen domain containing (ADIPOQ), immunoglobulin kappa constant (IGKC), myeloperoxidase (MPO), proteasome 20S subunit alpha 2 (PSMA2), actin beta (ACTB), protein S100 A9 (S100A9), vitamin D binding protein (GC), ferritin heavy chain 1 (FTH1), α2‐HS‐glycoprotein (AHSG), glyceraldehyde 3‐phosphate dehydrogenase (GAPDH), apolipoproteinC3 (APO C3), peptidylprolyl isomerase A (PPIA), retinol binding protein 4 (RBP4), keratin 10 (KRT10) (Table 1).

**Table 1:** Evaluation of biological characteristics of 31 proteins.

Symbol	Cellular component	Molecular function	Biological process	Reference
ACTB	– Membrane – Extracellular tissue and exosome – Filament – Cytoplasm	– Involved in cytoskeleton maintenance – ATP binding – Protein kinase binding	– Cell movement – Neuron generation and elongation – Cytoskeleton organization	[126]
AHSG	– Platelet granule – Endoplasmic reticulum lumen – Extracellular region	– Kinase inhibitor activity – Endopeptidase inhibitor activity	– Inflammation regulatory – Insulin regulatory signaling pathway – Regulation of bone mineralization – Pinocytosis – Skeletal system development	[127]
ALB	Cytoplasm	– Plasma colloid osmotic pressure – Endothelium regulator	– Transporter – Binding protein	[128]
		Banding and transporter		
APOA4	– Extracellular region – HDL particles – Plasma membrane – Cytosol – Endoplasmic reticulum lumen	– Protein binding – Cholesterol transfer – Amyloid beta binding – Lipid binding	– Cholesterol transport – Lipid metabolism – Protein Stabilization – Regulation of signaling pathway	[129, 130]
APOC3	– Extracellular region – Endosome	– Lipase inhibitor activity – Protein binding – Enzyme binding – Cholesterol binding	– Lipid metabolism – Lipid transporter – Regulation of signaling pathway	[131]
C3	– Extracellular region – Plasma membrane – Endoplasmic reticulum lumen – Cell surface – Azurophil granule lumen	– Endopeptidase inhibitor activity – Protein binding – Anaphylatoxin	– Immune response – Signaling pathway regulation – Regulation of phagocytosis – Fatty acid metabolism	[132]
FTH1	– Cytosol – Cytoplasm – Nucleus – Extracellular region	– Ferrous and ferric iron binding – Protein binding – Ferroxidase activity	– Cellular iron ion homeostasis – Immune response – Regulation of proliferation	[133]
GAPDH	– Nucleus – Cytosol – Cytoplasm – Microtubule cytoskeleton – Plasma membrane – Extracellular region	– Protein binding – NADP binding – NAD binding	– Microtubule cytoskeleton organization – Cytokine production – Glucose metabolic process – Immune response	[134]
GC	– Blood micro particle – Cytosol – Extracellular region – Lysosomal lumen – Cytoplasm	– Actin binding – Vitamin transmembrane transporter activity – Vitamin D binding	– Vitamin transport	[135, 136]
GSTP1	– Cytosol – Extracellular region – Nucleus – Cytoplasm – Plasma membrane	– Glutathione transferase activity – Protein binding – Nitric oxide binding	– Response to reach – Central nervous system development – Immune response – Regulation of cell signaling pathway	[137, 138]
HSPB1	– Extracellular region – Nucleus – Cytoplasm – Cytosol – Plasma membrane	– RNA binding – Protein binding	– Regulation of protein phosphorylation – response to unfolded protein – Involved in immune response	[139]
IGKC	– Plasma membrane – Extracellular region – Blood microparticle	– Immunoglobulin receptor binding – Antigen binding	– Immune response – B cell activation – Complement activation, classical pathway	[140]
KRT10	– Nucleus – Cytosol – Cell surface	– Protein binding – Structural constituent of skin epidermis	– Peptide cross‐linking – Keratinocyte differentiation	[141]
KRT9	– Nucleus – Cytosol – Extracellular region	– Structural constituent of cytoskeleton	– Spermatogenesis – Epidermis development – Skin development	[142]
LCN1	– Extracellular region	– Small molecules binding – Protein binding – Signaling receptor binding	– Sensory perception of taste – Proteolysis – Response to stimulus	[143, 144]
PSMA2	– Nucleus – Cytosol – Extracellular region – Nucleoplasm	– Protein binding	– Proteasomal protein catabolic process – Response to infection	[145]
RBP4	– Extracellular region	– Protein binding – Retinol binding	– Eye development – Gluconeogenesis – Organ and tissue development	[146]
S100A8	– Cytosol – Extracellular region – Nucleoplasm	– Microtubule binding – Calcium binding – Toll‐like receptor binding	– Autophagy – Apoptotic process – Wound healing – Astrocyte development	[147]
S100A9	– Cell junction – Extracellular region – Plasma membrane – Nucleus – Cytoplasm	– Calcium binding – Protein binding	– Autophagy – Apoptotic process – Wound healing – Astrocyte development – Inflammation response	[147, 148]
TF	– Clathrin‐coated endocytic vesicle membrane – Extracellular region – Vesicle	– Protein binding – transferrin receptor binding	– Osteoclast differentiation – Regulation of bone resorption – Signaling pathway regulation	[149]
UBE2N	– Extracellular region – Plasma membrane – Nucleus – Cytoplasm	– RNA binding – Protein binding – Ubiquitin conjugating enzyme activity	– Protein ubiquitination – Positive regulation of histone modification – Immune response regulation – Cell signaling pathway regulation	[150, 151]
HP	– Extracellular region – Blood micro particle – Haptoglobin‐hemoglobin complex	– Hemoglobin binding – Protein binding – Antioxidant activity	– Defense response – Acute phase protein – Positive regulation of cell death – Cellular oxidant detoxification	[152, 153]
EIF5A	– Cytoplasm – Cytosol – Nucleus – Membrane – Endoplasmic reticulum	– Protein binding – RNA binding – Ribosome binding	– regulation of apoptosis pathway – response to virus infection – regulation inflammation pathway	[154, 155]
APOC1	– Extracellular component – Endoplasmic reticulum	– Fatty acid binding – Protein binding – Phospholipase inhibitor activity	– Involved in lipid metabolism	[156]
ANG	– Nucleus – Extracellular component	– Protein binding – RNA binding – DNA binding – Heparin binding	– Angiogenesis – Cell migration – Cell communication	[157]
FGA	– Extracellular component – Plasma membrane – Platelet alpha granule	– Protein binding – Metal ion binding	– Fibrinolysis – Platelet aggregation	[158]
CRP	– Extracellular component	– Choline binding – Calcium ion binding – Protein binding – Protein binding – Complement binding	– Acute phase response – Inflammatory response – Innate immune response – Opsonization	[159]
MPO	– Cytoplasm – Membrane	– Oxidoreductase	– Inflammatory response – Immune response	[160]
FCN2	– Extracellular component – Cytoplasm	– Complement activation	– Inflammatory response – Immune response	[161]
ADIPOQ	– Extracellular component – Cellular surface	– immunomodulation	– Fatty acid metabolism – Inflammation response	[162]
PPIA	– Extracellular component – Cytoplasm	– RNA binding – DNA binding	– Positive regulation – Cell signaling pathway	[163]

## 4 DISCUSSION

This systematic review aimed to evaluate the studied protein markers using proteomics methods in patients with RD. Although systematic studies have been performed on the diagnostic and prognostic values of protein markers in RD, including COPD and Asthma, separately, no study has systematically evaluated common protein markers in RD. For the first time, this study assessed the protein markers identified by proteomics in COPD, Asthma, IPF, BO, and chemical warfare victims exposed to mustard gas in a systematic review.

Because identifying only two proteins in BO removed this disease from the results of the evaluation of common proteins between conditions. Therefore, in this section, the proteins of COPD, Asthma, IPF, and chemical warfare victims exposed to mustard gas are discussed. In this study, after removing duplications, identified 13,806 proteins in five diseases. After evaluation, 31 proteins were present in five conditions in the results section. After evaluating 31 proteins, we found that most are involved in the pathogenesis of inflammation and coagulation. Biological properties and their functions under normal conditions inside the cell are listed in Table 1.^126^, ^127^, ^128^, ^129^, ^130^, ^131^, ^132^, ^133^, ^134^, ^135^, ^136^, ^137^, ^138^, ^139^, ^140^, ^141^, ^142^, ^143^, ^144^, ^145^, ^146^, ^147^, ^148^, ^149^, ^150^, ^151^, ^152^, ^153^, ^164^

Inflammation is one of the primary pathogens of RD; most of the 31 common proteins in cited diseases were involved in inflammatory reactions. C3 is one of the components of complement that plays a vital role in the immune system, especially innate immunity. Studies have shown that C3 expression increases in some diseases, especially COPD and chemical warfare victims exposed to mustard gas.^165^, ^166^ C3 activates the NF‐κB pathway, which ultimately triggers the nucleotide‐binding oligomerization domain, leucine‐rich repeat, and pyrin domain containing (NLRP); it also induces inflammatory cytokines production, including IL‐1. It has also been shown that increased C3 expression in COPD leads to netosis.

Netosis is when neutrophils transfer their contents outward, creating a network that leads to the accumulation of immune cells and inflammation. Netosis increases TLR4 expression, which ultimately increases platelet activation. In addition, TLR4 activates NADPH Oxidase and increases ROS production by activating ERK.^167^, ^168^, ^169^ Increased ROS production causes neutrophil death and ketosis. Increased production of inflammatory cytokines can differentiate T helper2 (Th2) cells, which produce IL‐13 and IL‐5, leading to allergic reactions in patients, especially asthma patients.^168^

On the other hand, C3 has been shown to inhibit the lipoxin A4 (LXA4) expression by activating the MAPK/ERK pathway. LXA4 has been shown to regulate pulmonary epithelial cells by regulating tight junctions and preventing inflammation. However, LXA4 reduces tissue factor (TF) expression by inhibiting the TNF‐α/PI3K/AKT pathway.^170^, ^171^, ^172^, ^173^, ^174^ TF is the starting factor in the coagulation cascade, the word of which can cause thrombosis. Increased C3 expression in IPF leads to activation of the TGF‐β/SMAD pathway, which ultimately causes lung fibrosis.^175^, ^176^ Therefore, targeting C3 in mentioned diseases can prevent inflammation and disease progression.

Apolipoprotein A‐1 (ApoA1) is a component of HDL that is involved in cardiovascular disease prevention by regulating blood cholesterol levels. However, ApoA1 has also been shown to have an anti‐inflammatory role. It acts as a protective agent in preventing the progression of many diseases, including RD. In Asthma, APOA1 increases TGF‐β expression. Studies have shown that TGF‐β causes inflammation and airway obstruction by activating actin in epithelial cells.^177^ On the other hand, TGF‐β stimulates epithelial‐mesenchymal transition (EMT) to produce inflammatory mediators, including cytokines; they can affect pulmonary epithelial cells’ function.^178^, ^179^, ^180^, ^181^

However, ApoA1 in IPF has been shown to prevent inflammation by inhibiting TGF‐β expression. ApoA1 also inhibits inflammation and inflammatory cytokines production by inhibiting NF‐κB. In COPD, APOA1 expression has been shown to reduce the production of inflammatory cytokines, including IL‐1 and TNF‐α.^182^ Given that APOA1 suppresses NF‐κB can hypothesize that in COPD, APOA1 may reduce inflammatory cytokines production and inhibit inflammation by suppressing NF‐κB expression.

NF‐κB expression increases ferritin expression. Ferritin is one of the acute phase proteins that consists of two light and heavy chains. Asthma has been shown to reduce the expression of heavy‐chain ferritin (FTH1), which increases inflammation and oxidative stress. Therefore, it has been demonstrated that MAPK/ERK/c‐jun pathway increases FTH1 expression.^183^ However, this pathway is suppressed in Asthma. On the other hand, since TGF‐β expression has been shown to suppress the MAPK/ERK/c‐jun pathway, targeting TGF‐β in asthma patients can increase FTH1 expression and reduce inflammation and oxidative stress.^184^, ^185^, ^186^

**Figure 2:**
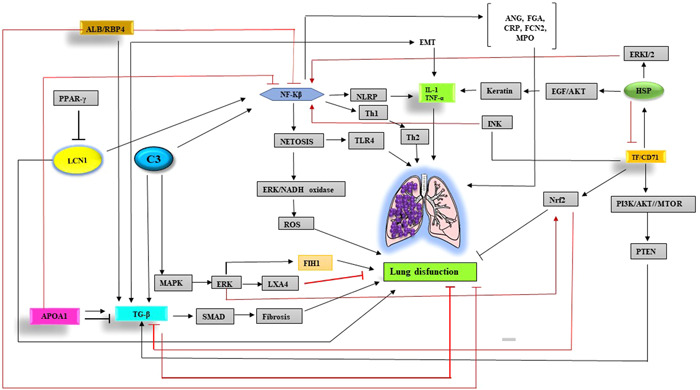
Evaluation of pathways involved in lung dysfunction: C3 activates the NF‐κB pathway and also induces TGF‐β expression, which ultimately causes inflammation and pulmonary fibrosis. C3 also causes Netosis and TLR4 expression. In addition, it induces LXA4 expression through the MAPK/ERK pathway, which prevents inflammation. TF/CD71 acts as a double‐edged sword, causing inflammation on the one hand and preventing cell damage by expressing Nrf2 on the other. ALB, albumin; APOA1, Apo; Apo lipoprotein A1; EMT, epithelial mesenchymal transition; HSP, heat shock protein; LCN1, lipocalin 1; LXA4, lipoxin A4; NF‐κB, nuclear factor kappa B; NLRP, nucleotide‐binding oligomerization domain, leucine rich repeat and pyrin domain containing; Nrf2, NF‐E2–related factor 2; PPARγ, peroxisome proliferator‐activated receptor gamma; RBP4, retinol binding protein 4; ROS, reactive oxygen species; TF, tissue factor TGF‐β, tumor growth factor‐β; Th, T helper; TLR, toll like receptor.

Studies have shown that increased ferritin expression in IPF, unlike Asthma, is associated with a poor prognosis leading to increased pulmonary fibrosis.^187^ It has also been shown that increased ferritin expression in COPD can be related to airway obstruction and disease progression (Figure 2).^188^

Transferrin (TF) is another factor that plays a vital role in the pathogenesis of RD, the same as ferritin. Accordingly, it has been shown that in asthma and COPD, increased TF expression leads to TLR2 activation and, ultimately, inflammation. CD71 is known as the TF receptor. Heat shock protein (HSP) in COPD reduces inflammation by inhibiting the CD71/JNK/NF‐κB pathway. HSP also increases keratin expression and inflammation through the EGF/AKT pathway. In asthma, HSP causes inflammation by activating the ERK1/2/NF‐κB pathway. In IPF, after TF binding to CD71, the PI3K/AKT/mTOR/PTEN pathway is activated, which increases TGF‐β expression and causes pulmonary fibrosis.^189^, ^190^, ^191^, ^192^, ^193^, ^194^ While shown in the mustard gas‐exposed patient, TF has a protective role.

TF reduces ROS production and inflammation. Therefore, can hypothesize that TF may prevent cell damage in patients by expressing Nrf2, which has an antioxidant role.^195^ On the other hand, HSP has been shown to cause pulmonary fibrosis by activating the TGF‐β/SMAD pathway.^196^, ^197^ HSP acts as a double‐edged sword and plays an influential role in the occurrence and inhibition of inflammation; it is possible to control the progression of inflammation and improve patient survival by identifying its upstream and downstream pathways (Figure 2).

Lipocalin1 (LCN1) is another molecule like TF; it has a protective role. In COPD, LCN1 expression increased, associated with inflammation and oxidative stress prevention. LCN1 has been shown to increase the expression of Nrf2 and HO‐1 by activating the MAPK/ERK pathway. These two factors are antioxidants and prevent cell damage.^198^ LCN1, like COPD, plays a protective role in the mustard gas‐exposed patient and protects cells against ROS.^199^, ^200^ In contrast, increased LCN1 expression in asthma has been shown to activate the NF‐κB pathway and causes inflammation.^201^ In IPF, increased LCN1 expression activates the NF‐κB pathway through MMP‐9. However, LCN1 expression in IPF is reduced by Peroxisome proliferator‐activated receptor gamma (PPAR‐γ) (Figure 2).^202^

Retinol binding protein 4 (RBP4) is one of the genes involved in the pathogenesis of RD and regulates TGF‐β. In COPD, decreased RBP4 expression is associated with increased disease progression. Albumin (ALB) stabilizes by binding to RBP4. ALB/RBP4 then reduces inflammation by inhibiting NF‐κB. In addition, it blocks the TGF‐β/SMAD signaling pathway.^203^ In asthma, decreased RBP4 expression is associated with increased inflammation.^204^ In IPF, TGF‐β increases the expression of proteasome 20S subunit alpha 2 (PSMA2), leading to inflammation and pulmonary fibrosis. In asthma, PSMA2 expression causes inflammation by activating the NF‐κB and producing inflammatory cytokines.^205^ In COPD, PSMA2 activates NF‐κB and caspase3, causing lung cell apoptosis and worsening patients' clinical course. Thus, targeting RBP4 prevents inflammation and reduces apoptosis (Figure 2).^206^

In general, in RDs, increased expression of inflammatory genes causes exacerbation of disease. Activation of NF‐κB lead to the production of cytokine by immune cell and induce activation of inflammation signaling pathway. The previous studies showed that activation of NF‐κB leads to increased serum level markers, including CRP, MPO, ANG, FGA, and FCN2.^207^, ^208^, ^209^, ^210^

The strengths of this study are as follows: This study focuses on the proteomic analysis of five diseases, namely COPD, IPF, asthma, chemical warfare victims exposed to mustard gas, and BO. It adopts a systematic review approach, which is the first of its kind. Furthermore, common proteins among the investigated diseases have been identified, which can be potentially utilized in the future for designing appropriate therapeutic strategies.

The limitation of the study are as follows: The methods used in the reviewed articles mostly consist of high dropout. Also, the study did not include an experimental validation of the identified proteins, which would provide more evidence for their potential role in disease pathogenesis.

## 5 CONCLUSION

Considering that 31 proteins (TF, GSTP1, UBE2N, HP, EIF5A, KRT9, APO A4, ALB, APO C1, ANG, S100A8, FGA, HSPB1, C3, LCN1, CRP, FCN2, ADIPOQ, IGKC, MPO, PSMA2, ACTB, S100A9, GC, FTH1, AHSG, GAPDH, APO C3, PPIA, RBP4, and KRT10) are common in five diseases, identifying their upstream and downstream pathways can be essential in designing treatment strategies and managing patients.

## 6 FUTURE PERSPECTIVE

It would be better to conduct experimental studies on human samples to confirm the results of this study. Additionally, examining differential proteins in each of the diseases and conducting additional studies on other OMICs, including transcriptome and metabolome, and integrating their data with proteome, can provide a better understanding of the conditions.

## AUTHORS CONTRIBUTIONS

B. Fatemeh Nobakht M. Gh has conceived the manuscript and revised it. Hadi Rezaeeyan, B. Fatemeh Nobakht M. Gh, and Masoud Arabfard extraction data and analysis. Hamid R. Rasouli and Alireza Shahriary design strategy search. All authors read and approved the final manuscript.

## CONFLICT OF INTEREST STATEMENT

The authors declare no conflict of interest.

## ETHICS STATEMENT

This article does not contain any studies with human participants or animals performed by any of the authors.

## Supporting information

Supporting information.

## Data Availability

The datasets used analyzed during the current study are available from the corresponding author upon reasonable request.
