# Supplementary material for: Evaluation of common protein biomarkers involved in the pathogenesis of respiratory diseases with proteomic methods: A systematic review
Source: Immun Inflamm Dis. 2023 Nov 20;11(11):e1090. doi: 10.1002/iid3.1090 (PMC10659759; doi:10.1002/iid3.1090)
Supplement: Supplementary file 1 — Supporting information. [file IID3-11-e1090-s001.docx]

Table 1. Summary detail information of study related to COPD.

| **Author (year)** | **Sample size** | **Country** | **Male/Female** | | **Mean age ± SD** | | **Biological specimens** | **Technological platform used** | **Statistical analysis** | **Proteomic markers (UP/Down regulation)** | **Model characteristics** | **Validation** | **Final finding** | **Ref** |
| --- | --- | --- | --- | --- | --- | --- | --- | --- | --- | --- | --- | --- | --- | --- |
|  |  |  | **Case** | **Control** | **Case** | **Control** |  |  |  |  |  |  |  |  |
| Kononikhin et al. 2015 | 17 COPD  23 Control | Russian | 13  4 | 10  13 | 64.7±4.7 | 27.5±4.8 | exhaled breath condensate | HPLC-MS/MS | ------------ | 16 proteins UP-regulation and 10 proteins Down regulation in patient compare to Control group | ------------- | ----------- | Analysis of EBC samples by HPLC-MS/MS can useful for detection potential protein biomarker inflammation in COPD patients | (1) |
| Bhowmik et al. 2019 | 5 COPD  5 Control | India | 5  0 | 3  2 | 62(55-70) | 34(26-44) | Plasma | SDS PAGE, ELISA, LC-MS/MS | T test | IL-33 UP-regulation and Apo E Down regulation in patient compare to control groups | -------------------- | Western Blot | Evaluation of protein expression can useful for prognostic marker for disease | (2) |
| Ishikawa et al. 2010 | 4 COPD  4 Control | Finland | 1  3 | 3  1 | 58±4 | 59±7 | Sputum, Tissue, Bronchoalveolar lavage fluid | 2DE, LC/MS-MS, Immunohistochemistry, Chromatography | Mann-Whitney U and Kruskal Wallis test | Hb A and Hb B monomer Down but Hb A and Hb B complex UP-regulation in patient compare to control group | -------------- | Western Blot | Expression of hemoglobin in IPF declined | (3) |
| Loi et al. 2017 | 41 COPD  7 Control | Netherland | 30  11 | 5  2 | 60±8 | 60±6 | Peripheral blood (neutrophils) | 2D-DIGE | T tests, ANOVA | 7 proteins UP-regulation and 4 proteins Down regulation in patient compare to Control group | ---------------- | ------------ | Evaluation inflammation can useful for diagnosis of COPD patients | (4) |
| Baralla et al. 2018 | 29 Mild COPD  43 Control | Italy | ------- | ----- | 74.8±5.9 | 74.8±5.9 | Plasma | MALDI TOF-MS, 2DE | T test | 9 proteins UP-regulation and 3 proteins Down regulation in patient compare to Control group | --------------- | western blotting | Monitoring change protein expression in COPD patients can useful for design prevention strategy and proper treatment | (5) |
|  | 14 Moderate COPD  43 Control |  |  |  |  |  |  |  |  |  |  |  |  |  |
| Linja-AHO et al.2013 | 12 COPD stage I-II  13 Control | Finland | 7  5 | 7  6 | 61 | 65.5 | Sputum and Plasma | Western blot , ELISA, Immunohistochemistry | ANOVA, Kruskal–Wallis, Mann–Whitney, Spearman correlation coefficient, scatter plots | AAT UP-regulation in patient compare to Control group | ----------------- | ------------- | Increased AAT in COPD associate with progression disease and chronic exposure to smoke | (6) |
|  | 7 COPD stage IV  13 Control |  | 4  3 | 7  6 | 57 | 65.5 |  |  |  |  |  |  |  |  |
| Casado et al.2007 | 15 COPD  7 Control | Italy | 11  4 | 5  2 | 70±9 | 68±2 | Sputum | CapLC-ESI-Q-TOF | Paired student t test | 5 proteins UP-regulation and 6 proteins Down regulation in patient compare to Control group | -------------- | ------------ | Use of proteomic methods can useful for detection protein biomarker related COPD stages | (7) |
|  | 10 COPD& emphysema  7 Control |  | 8  2 | 5  2 | 69±8 | 68±2 |  |  |  |  |  |  |  |  |
| Nicholas et al. 2010 | 16 COPD stage I  20 Control | UK | 11  6 | 6  14 | 57.9 (52.4–63.4) | 52.9 (47.3–56.4) | Sputum | 2DE, MS/MS, Immunohistochemistry, | t test, Mann-Whitney U and Kendall’s tau b | apolipoprotein A1 (Down), lipocalin-1 (Down) | ----------------- | Western blot , ELISA | Reduced expression of apolipoprotein A1 and lipocalin-1 caused susceptibility COPD patients to infection | (8) |
|  | 25 COPD stage II  20 Control |  | 18  7 | 6  14 | 60.3 (52.6–67.7) | 52.9 (47.3–56.4) |  |  |  |  |  |  |  |  |
|  | 3 COPD stage III  20 Control |  | 2  1 | 6  14 | 61.2 (57.6–70.8) | 52.9 (47.3–56.4) |  |  |  |  |  |  |  |  |
| Titz et al. 2015 | 60 COPD  60 Control | Switzerland | 40  20 | 38  42 | 57.17±7.16 | 55.46±7.45 | Sputum | LC-MS/MS | logistic lasso regression, linear discriminant analysis, k-fold cross validation Fisher test | 5 proteins UP-regulation and 8 proteins Down regulation in patient compare to Control group | ------------------- | ------------ | Exposure COPD patient to smoke cause change expression proteins related oxidative/ protease | (9) |
| Alexandre et al. 2012 | 25 COPD  28 Control | USA | 17  8 | 13  15 | 61±11 | 45±12 | Peripheral blood | strong cation exchange (SCX) chromatography, RPLC–MS/MS, | -------------- | 10 proteins UP-regulation and 3 proteins Down-regulation in patient compare to Control groups | ----------------- | Western blot | Reduced expression of VPS13A and CYB5R3 in COPD associate with deformity of erythrocyte membrane and high risk to developing methemoglobinemia | (10) |
| Sun et al. 2019 | 19 COPD  19 Control | China | 16  3 | 14  5 | 64.0±6.9 | 59.1±9.4 | Exhaled breath condensate | Tandem mass tags, LC-MS/MS | T test, chi-square test, Fisher's exact test and Two-tailed test | 9 proteins Down-regulation and 15 proteins UP regulation in patient group compare control group | ------------------ | --------------- | Use of TMT method suitable for proteomic analyzing EBC samples for detection biomarkers | (11) |
| Tu et al. 2014 | 10 COPD  10 Control | USA | 7  3 | 6  4 | 67.8±8.5 | 63.4±11.73 | bronchoalveolar lavage fluid, | Nano RPLC/MS | Pearson correlation coefficient | 50 proteins UP-regulation and 26 proteins Down regulation in patient compare to Control group | --------------- | Western blot | Detection of biomarker by proteomic methods in COPD patient can useful for diagnosis change protein expression | (12) |
| Brandesma et al. 2019 | 10 COPD  8 Control | Netherland | 2  8 | 4  4 | 58 | 65 | Lung tissue | LC-MS/MS, Chromatography, | ANOVA, Linear regression, Mann-whitney, | 177 proteins UP-regulation and 150 proteins Down regulation in COPD compare to Control group |  |  | Use of proteomic method can useful for diagnosis of pathogenesis of COPD | (13) |
| Merkel et al. 2005 | 145 COPD  380 Control | Germany | ------- | ------ | ----- | ---- | Bronchoalveolar lavage fluid | SELDI-MS, LC-MS/MS, HPLC, nanoLC-MS/MS | ------------ | 10 proteins UP-regulation in patient compare to Control group | ----------------- | -------------- | Use of proteomic method can help for diagnosis biomarker related to COPD | (14) |
| Shahriary et al.2015 | 10 COPD  10 Control | IRAN | 10  0 | 10  0 | 60.08±2.34 | 54.4±2.9 | Blood neutrophil | 2D -SDS PAGE, MALDI-TOF-MS/MS | ANOVA, Tukey’s range test, post-hoc analysis | 7 proteins protein UP-regulation and 6 protein Down regulation in patient compare to control | ------------------- | -------------- | Apart of inflammation and oxidative stress , imbalance of protease and anti-protease involved in pathogenesis of COPD patients | (15) |
| Tan et al. 2020 | 20 stable COPD  20 Control | Australia | ------- | ------ | ----- | ---- | Plasma | iTRAQ , MRM, HPLC | t-test, Mann–Whitney test, | 6 proteins UP-regulation and 3 proteins Down-regulation in in AE COPD compare to Control while 6 proteins UP-regulation and 3 proteins Down-regulation in in stable COPD compare to Control | ---------------- | ELISA | Detection protein biomarkers in COPD needs to validation for use in diagnosis and management severity of disease | (16) |
|  | 20 AECOPD  20 Control |  | ------- | ------ | ----- | ---- |  |  |  |  |  |  |  |  |
| Ahrman et al.2018 | 5 COPD  5Control | Sweden | 1  4 | 4  1 | 60(52-69) | 55(39-65) | Lung Tissue | LC-MS/MS, SDS-PAGE, Immunohistochemistry | Kruskal-Wallis test | 13 proteins UP-regulation and 12 proteins Down regulation in patient compare to Control group | ----------------- | -------------- | Imbalance between protease-protease inhibitor resulting impairing tissue remodeling and cause emphysema in COPD patients | (17) |
| Lee et al. 2009 | 7 COPD  8 Control | Seoul | 7  0 | 4  4 | 56.0±9.3 | 59.0±8.1 | Lung Tissue | 2DE, MALDI-TOF-MS | Kruskal-Wallis test, Wilcoxon rank-sum test | 8 proteins UP-regulation in patient compare to Control Groups | ------------------ | Western blot, Immunohistochemistry | Increased expression of MMP13 in thioredoxin-like 2 in alveolar macrophages and type II pneumocytes cause airflow limitation and development COPD | (18) |
| Li et al.2020 | 26 Quick COPD  25 Control | China | 22  4 | 13  12 | 67.73±8.43 | 61.57±9.31 | Plasma | iTRAQ, LC-MS/MS | Gene ontology by AgBase database, Protein-protein interaction analysis by database STRING | 9 proteins UP-regulation and 6 proteins Down regulation in patient compare to Control group | ------------------- | -------------- | Compare protein biomarkers between COPD and Control can useful for diagnosis biomarker related to development of COPD | (19) |
|  | 26 Slow COPD  25 Control |  | 16  10 | 13  12 | 68.58±8.26 | 61.57±9.31 |  |  |  |  |  |  |  |  |
|  | 25 Normal COPD  25 Control |  | 15  10 | 13  12 | 66.88±10.83 | 61.57±9.31 |  |  |  |  |  |  |  |  |
| Chen et al. 2012 | 7 AECOPD  5 Control | China | ------- | ------ | ----- | ---- | Plasma | Multiplex antibody array | T test, Linear regression | 28 proteins UP-regulation and 2 proteins Down regulation in COPD compare to Control group | -------------------- | ELISA | Use of proteomic method can useful for diagnosis biomarkers related to COPD disease | (20) |
|  | 5 COPD  5 Control |  | ------- | ------ | ----- | ---- |  |  |  |  |  |  |  |  |
| Chen et al. 2012 | 6 AECOPD  6 Control | China | 3  3 | 3  3 | 69.0±1035 | 6117±9.16 | Plasma | Microarray assay | ANOVA, Spearman correlation test, T test | 2 proteins UP-regulation and 18 proteins Down regulation in patient compare to Control group | ------------------ | -------------- | Evaluation expression of protein biomarkers can useful for diagnosis disease stage | (21) |
|  | 6 COPD  6 Control |  | 3  3 | 3  3 | 61.50±8.43 | 61.17±9.16 |  |  |  |  |  |  |  |  |
| Baraniuk et al.2015 | 15 COPD  7 Control | USA | 11  4 | 5  2 | 70.0±9.0 | 68.0±2.0 | Sputum | MS/MS | ANOVA, T test, Linear regression | 17 proteins UP-regulation and 11 proteins Down regulation in patient compare to Control group | ------------------ | -------------- | Evaluation biomarkers useful for determine phenotype pathogenesis marker related to emphysema and COPD | (22) |
|  | 10 COPD& Emphysema  7 Control |  | 8  2 | 5  2 | 69.0±8.0 | 68.0±2.0 |  |  |  |  |  |  |  |  |
| Gao et al.2015 | 122 COPD  62 Control | Finland | 96  16 | 20  42 | 60.45 | 55.6 | Lung Tissue, Sputum | MS/MS, ELISA, Western blot, 2DE, Immunohistochemistry | Kruskal–Wallis test, Mann–Whitney, χ2 test, Spearman correlation test, ANOVA, T test | Expression of vitamin D binding protein increased in patient compare to Control group | ------------------ | -------------- | Elevation expression of vitamin D binding protein associated with airway obstruction | (23) |
| Bandow et al. 2008 | 24 COPD  24 Control | Germany | 24  0 | 24  0 |  |  | Plasma | SDS-PAGE, 2DE, MALDI-MS | T test, ANOVA, Principal component analysis | 5 Proteins UP-regulation and 16 Proteins Down regulation in patient compare to control groups | ------------------ | -------------- | Evaluation of retinal-binding protein (RETB) and fibrinogen can use for monitoring of COPD patients | (24) |
| Gao et al.2020 | 21 COPD stage I  32 Control | Japan | 18  3 | 12  20 | 55.1 | 54.9 | Plasma, Sputum | MS/MS, ELISA, Western blot, 2DE | Kruskal–Wallis test, Mann–Whitney, χ2 test, Spearman correlation test, | Expression of vitamin D binding protein increased in patient compare to Control group | ------------------ | -------------- | Elevation expression of vitamin D binding protein associated with airway obstruction | (25) |
|  | 23 COPD stage II  32 Control |  | 20  3 | 12  20 | 62.7 | 54.9 |  |  |  |  |  |  |  |  |
| López-Sánchez et al. 2017 | 46 COPD  49 Control | Spain | 42  4 | 38  11 | 66±7 | 60±9 | Exhaled breath condensate | LC-MS/MS, MS/MS | ANOVA, Levene , Shapiro-Wilk, Kruskal-Wallis test | 28 proteins UP-regulation and 2 proteins Down regulation in patient compare to Control group | ROC curve, Specify, Sensitivity | ------------- | Proteomic analysis of EBC samples is appropriate method for determine biomarker for diagnosis of lung disease | (26) |
| Francioci et al. 2014 | 5 COPD  5 Control | Netherland | 0  5 | 1  4 | 66 | 49 | Epithelial lining fluid | iTRAQ, MALDI-TOF | Mann-Whitney U test, Wilcoxon test, | Result showed that expression of 24 proteins in COPD and 7 proteins in Control group changed | ------------------- | Immunohistochemistry, ELISA | Results showed that SerpinB3 and Uteroglobin useful for understand pathogenesis of COPD | (27) |
| Francioci et al. 2013 | 4 COPD  4 Control | Netherland | 4  0 | 2  2 | 67 (47-74) | 61.5 (45-83) | Epithelial lining fluid | SDS-PAGE, chipLC-MS/MS, Immunohistochemistry, MALDI-TOF, nano LC-MS/MS | T test | 46 proteins UP-regulation and 30 proteins Down regulation in patient compare Control group | ------------------ | -------------- | Use of Epithelial Lining Fluid can proper for diagnosis change expression of proteins in COPD | (28) |
|  | 4 COPD  4 Control |  | 2  2 | 2  2 | 60.5 (53-66) | 57.5(20-87) |  |  |  |  |  |  |  |  |
|  | 5 current COPD  5 Control |  | 5  0 | 1  4 | 69 (44-72) | 65 (36-70) |  |  |  |  |  |  |  |  |
|  | 5 ex-smoker COPD  5 Control |  | 5  0 | 1  4 | 72.5 (65-76) | 65 (36-70) |  |  |  |  |  |  |  |  |
| Pastor et al. 2013 | 15 COPD  15 Control | Spain | 15  0 | 15  0 | 61.5 | 61.3 | bronchoalveolar lavage | 2DE-DIGE, MALDI-TOF/TOF | -------------- | 33 proteins UP-regulation while 7 proteins Down regulation in patient compare Control group | ------------------- | Western blot | Protein profiles useful for elucidate pathogenesis of COPD and diagnosis progression of COPD to lung cancer | (29) |
|  | 15 LC&COPD  15 Control |  | 15  0 | 15  0 | 60.7 | 61.3 |  |  |  |  |  |  |  |  |
| Saleem et al. 2019 | 100 COPD  50 Control | Pakistan | 100  0 | 50  0 | 20-80 | 20-80 | Plasma | One and 2DE-SDS-PAGE. MALDI-TOF-TOF-MS, Affinity chromatography | -------------- | 7 proteins increased while one protein Down regulation in patient compare Control group | ------------------ | -------------- | Diagnosis of protein biomarkers can use for progression COPD progression to lung cancer | (30) |
| Fumagalli et al. 2012 | 15 COPD  25 Control | Italy | 7  8 | 12  13 | 65±8.0 | 33±4.5 | exhaled breath condensate | LC-MS/MS, SELDI-MS, SDS-PAGE, | MASCOT, Bland-Altman model | 26 proteins Down regulation in patient compare Control group | -------------------- | Western blot, SELDI-TOF | Detection and monitoring protein biomarker useful for discriminate COPD from control | (31) |
| Kohler et al. 2013 | 15 Smoker COPD  23 Control | Germany | 8  7 | 10  13 | 57.5 | 55.5 | bronchoalveolar lavage | 2DE-DIGE, MS/MS | ANOVA, Chi-Square, T test | LMNA Down regulation and  LTA4H UP-regulation in patient compare to control group | R2, Q2 | Western blot | Detection biomarker proteins according to gender can useful for treatment COPD patients | (32) |
|  | 6 Exsmoker COPD  23 Control |  | 3  3 | 10  13 | 60.5 | 55.5 |  |  |  |  |  |  |  |  |
| Yang et al.2018 | 18 COPD with smoker  18 Control | Sweden | 10  8 | 9  9 | ----- | ---- | bronchoalveolar lavage | iTRAQ, LC-MS/MS | ANOVA, Pearson correlation coefficient, T test | 188 proteins changed expression between patient and control groups | R2, Q2 | ------------- | Results showed that dysregulation of several phagocytosis-related pathways in BAL COPD patients associate with severity | (33) |
|  | 8 ex-smoker COPD  18 Control |  | 3  5 | 9  9 | ----- | ---- |  |  |  |  |  |  |  |  |
| Verrills et al. 2011 | 5 COPD  17 Control | Australia | 2  3 | 8  9 | 65.7±10.6 | 44.2±14.4 | Platelet-depleted plasma | 2D-DIGE, MALDI-TOF/TOF | Logistic regression, T test, ANOVA, Spearman’s rank correlation | 8 proteins UP-regulation and 10 protein Down regulation in patient compare to Control group | Sensitivity, specificity, ROC curve | ELISA, Western Blot | Proteomic biomarkers showed that Iron metabolism and Acute phase response involved in pathogenesis of air way in COPD | (34) |
| Gomes-Alves et al 2010 | 16 COPD  54 Control | Portugal | 12  4 | 21  33 | 63.2±5.8 | 41.6±14.5 | Serum | SELDI-TOF | Logistic regression, Kruskal-Wallis, T test, Dunn's Multiple Comparison Test, | 6 proteins different expression between two groups | Sensitivity, specificity, ROC curve | -------------- | Evaluation of protein biomarker can useful for diagnosis and management of lung disease such as COPD | (35) |
| Langereis et al. 2011 | 13 COPD  6 Control | Netherlands | 11  2 | 5  1 | 65.5 | 60.2 | Peripheral Blood | 2DE | ANOVA, Mann-Whitney, T test, | Some proteins changed their expression between two groups | ---------------- | -------------- | Profile proteins changed in COPD compare to Control groups | (36) |
| Sun et al.2019 | 6 frequent exacerbators COPD  15 Control | China | 5  1 | 12  3 | 63.80±5.91 | 63.3±5.64 | Lung tissue | LC-MS/MS | ANOVA, Mann-Whitney, T test, Fisher’s exact test, Kruskal-Wallis H test, Chi-square test | 40 proteins UP-regulated and 27 proteins Down regulated in patients compared to the control groups | ---------------- | Western blot | Result shown that immune system involved in pathogenesis of COPD, and diagnostic biomarker can be useful to find treatment | (37) |
|  | 15 infrequent exacerbators COPD  15 Control |  | 12  3 | 12  3 | 63.80±5.51 | 63.3±5.64 |  |  |  |  |  |  |  |  |
| Gray et al.2008 | 24 COPD  20 Control | UK | 14  10 | 9  11 | 65.2 ± 1.2 | 36.4 ± 2.1 | Sputum | SELDI-TOF, ELISA | Mann-Whitney, T test | 3 proteins UP-regulated, and 2 protein Down regulated in patients compared to the control groups | ----------------- | -------------- | Use of proteomic methods can be useful to detect biomarkers related to inflammation in early stage of lung disease, such as COPD | (38) |
| Terracciano et al. 2011 | 6 COPD  5 Control | Italy | 4  2 | 3  2 | 69±7 | 55±21 | Sputum, Whole human saliva | MALDI-TOF/TOF MS, MALDI MS, | T test | 2 proteins UP-regulated in patients compared to the control group | ------------------ | -------------- | Use of proteomic methods can be useful for monitoring the inflammation in COPD by biomarkers | (39) |
| Hu et al. 2011 | 24 COPD smoker  24 Control | China | 16  8 | 19  5 | 63.7±5.4 | 61.9±6.9 | Lung tissue | 2-DE, MALDI-TOF, Immunohistochemistry | ANOVA, Newman- Keuls, Chi-Square | 22 proteins UP-regulated and 2 proteins Down-regulated in patients compared to the control groups | ------------------ | Western blot, Immunohistochemistry | Smoking causes increased expression of HSP-27 and CyPA, which are involved in COPD pathogenesis | (40) |
| Merali et al. 2014 | 20 COPD II  30 Control | USA | 20  0 | 30  0 | 65±1 | 64±1 | Plasma | GelC-MS/MS | ANOVA, Mann-Whitney, Multivariate linear regression, Neuman-Keuls, | 13 proteins UP-regulated and 18 proteins Down regulated in patients compared to the control group | ROC curve, Specify, Sensitivity | Western blot, ELISA | Detecting biomarkers by proteomic methods can be useful to determine the pathogenesis of COPD | (41) |
|  | 30 COPD III-IV  30 Control |  | 30  0 | 30  0 | 64±1 | 64±1 |  |  |  |  |  |  |  |  |
| Ohlmeier et al. 2008 | 7 COPD II-III  7 Control | Finland | 3  4 | 5  2 | 62.0±4.3 | 55.0±6.8 | Tissue and sputum | 2-DE, MS/MS | Mann-Whitney | SP-A expression increased in patients compared to the control group | ----------------- | Western blot, immunohistochemistry | SP-A can use as diagnostic biomarker for early stage of disease | (42) |
|  | 6 COPD IV  7 Control |  | 3  3 | 5  2 | 56.5±35 | 55.0±6.8 |  |  |  |  |  |  |  |  |
| Ohlmeier et al. 2016 | 32 COPD I  34 Control | Finland | 26  6 | 9  25 | 58.6±9.35 | 57.65±12.1 | Sputum, Plasma, Lung Tissue | 2D-DIGE, MALDI/TOF-TOF | Chi-Square, ANOVA, Kruskal-Wallis test, Mann-Whitney U-test | 6 protein UP-regulated in patients compared to the control group | ------------------ | Western blot, ELISA | Result showed that TGM2 can use as diagnostic biomarker, and also therapeutic target in COPD patients | (43) |
|  | 33 COPD II-III  34 Control |  | 26  7 | 9  25 | 60.5±6.8 | 57.65±12.1 |  |  |  |  |  |  |  |  |
| Ohlmeier et al. 2012 | 7 COPD  7 Control | Finland |  |  | 58 | 53 | Sputum, Plasma | 2D-DIGE/ MS | ANOVA, Mann-Whitney, Pearson’s correlation coefficient | 12 proteins UP-regulated and 4 Proteins Down-regulated in COPD compared to the control group |  | Western blot, ELISA, Immunohistochemistry | Increased expression of PIGR was associated with inflammation and related with pathogenesis of COPD | (44) |
| Ohlmeier et al. 2010 | 9 mild/moderate COPD  12 severe COPD  4 Control | Finland |  |  |  |  | bronchoalveolar lavage fluid, Tissue | 2DE, ELISA, | Mann–Whitney U | cRAGE down regulated in COPD patients compared to the control groups | ------------- | Western Blot | RAGE protein is involved in the pathogenesis of COPD | (45) |
| York et al. 2010 | 18 Slow decline COPD  18 Control | USA | 10  8 | 9  9 | 63.6 | 57.2 | Plasma | HPLC, LC-MS/MS | Regression coefficient | 4 proteins UP-regulated and 7 proteins Down-regulated in COPD compared to the control group | ---------------- | ---------------- | Evaluating the proteomics method can be useful to determine the pathogenesis of disease | (46) |
|  | 18 Rapid decline COPD  18 Control |  |  |  |  |  |  |  |  |  |  |  |  |  |
| Diao et al. 2016 | 53 COPD  33 Control | China | 53  0 | 33  0 | 64 ± 5.4 | 58 ± 5.1 | Plasma | LC-MS/MS, iTRAQ | Shapiro-Wilk (S-W) test, ANOVA, t-Test, Spearman correlation  Analyses, Multivariate linear regression | 15 proteins UP-regulated and 28 proteins Down-regulated in COPD compared to the control group | sensitivity , specificity , ROC curve | ELISA | Evaluation of the FETUB can be useful to diagnose COPD and manage the disease progression | (47) |
|  | 4 COPD  4 Control |  | 4  0 | 4  0 | 63 ± 5 | 62 ± 4 |  |  |  |  |  |  |  |  |
| Liu et al. 2020 | 3 COPD  3 Control | China | 3  0 | 1  2 | 57 | 79 | Lung tissue | HPLC, LC-MS/MS | T test | 55 proteins UP-regulated and 118 Proteins Down-regulated in COPD group compared to the control group | ------------- | Western blot | Down-regulation of GP6, PF4, and THBS1 proteins, which are needed for platelet activation and cause hemostasis disorders. Also, Down-regulation of CD163, MARCO and  VSIG4 causes inhibition regulation of inflammation, and COPD progression. | (48) |
| Xia et al.  2013 | 57 COPD  40 Control | China | 32  25 | 24  16 | 66 (51–85) | 64 (50–80) | Serum | SELDI-TOF-MS | Ciphergen protein chip Software 3.1.1., Wizard Software | 8 proteins UP-regulated and 12 proteins Down-regulated in COPD group compared to the control group | Specify, Sensitive, Accuracy rate and ROC curve | -------- | Using proteomic methods and diagnostic models can discriminate COPD patients from healthy control and can identify different stages of COPD | (49) |
|  | 30 Stable COPD  40 Control |  | 24  6 | 24  16 | 62 (51–76) | 64 (50–80) |  |  |  |  |  |  |  |  |
|  | 27 AE COPD  40 Control |  | 8  19 | 24  16 | 70 (56–85) | 64 (50–80) |  |  |  |  |  |  |  |  |

Abbreviation: COPD: Chronic obstructive pulmonary disease, 2DE: Two-dimensional gel electrophoresis, LC/MS-MS: Liquid Chromatography with tandem mass spectrometry, LC-MRM: Liquid Chromatography Multiple reaction monitoring, MALDI-TOF: MALDI coupled to time-of-flight, MALDI-ToF–ToF/MS: MALDI coupled to time-of-flight mass spectrometry, iTRAQ: Isobaric tags for relative and absolute quantitation; HPLC: High-performance liquid chromatography, SDS-PAGE: sodium dodecyl sulphate–polyacrylamide gel electrophoresis, ELISA: Enzyme-linked immunosorbent assay, HPLC: High-performance liquid chromatography, LC-IMS-MS: Liquid Chromatography-Ion mobility spectrometry–mass spectrometry, SELDI-TOF-MS: Surface enhanced laser desorption/ionization time-of-flight mass spectrometry, MRM: Multiple reaction monitoring.

Table 2. Summary detail information of study related to IPF.

| **Author (year)** | **Sample size** | **Country** | **Male/Female** | | **Mean age ± SD** | | **Biological specimens** | **Technological platform used** | **Statistical analysis** | **Proteomic markers (UP/Down regulation)** | **Model characteristics** | **Validation** | **Final finding** | **Ref** |
| --- | --- | --- | --- | --- | --- | --- | --- | --- | --- | --- | --- | --- | --- | --- |
|  |  |  | **Case** | **Control** | **Case** | **Control** |  |  |  |  |  |  |  |  |
| Ohlmeier et al. 2008 | 9 IPF  6 Control | Finland | 7  2 | 4  2 | 58.6 ± 3.8 | 58.0 ± 7.7 | Tissue and sputum | 2-DE, MS/MS | Mann-Whitney | One protein UP-regulated in patients compared to control group | ------------- | Western blot, immunohistochemistry | SP-A can use as a diagnostic biomarker early stage patients | (42) |
| Gao et al.2015 | 4 IPF  4 Control | Finland | 4  0 | 0  4 | 55.8 | 69.8 | Lung Tissue, Sputum | MS/MS, ELISA, Western blot, 2DE, Immunohistochemistry | Kruskal–Wallis test, Mann–Whitney, χ2 test, Spearman correlation test, ANOVA, T test | Expression of vitamin D binding protein increased in patients compared to the control group | ----------- | -------- | Vitamin D binding protein was associated with pathogenesis of disease | (23) |
| Foster et al.2015 | 4 IPF  5 Control | USA | 3  1 | 2  3 | 66.75 | 44.4 | bronchoalveolar lavage fluid | LC-MRM, LC/MS-MS, 2DE | Mann–Whitney U test, T test | 24 proteins UP-regulated and 25 proteins Down regulated in patients compared to the control groups | Roc curve, Specificity, Sensitive | ---------- | Use of proteomic methods help to determine the pathogenesis of IPF | (50) |
| Ishikawaet al. 2010 | 4 IPF  4 Control | Finland | 3  1 | 3  1 | 54.5±5 | 59±7 | Sputum, Tissue, Bronchoalveolar lavage fluid | 2DE, LC/MS-MS, Immunohistochemistry, Chromatography | Mann-Whitney U and Kruskal Wallis test | Hb A and Hb B monomer and complex Down regulated in patients compared to the control groups | --------------- | Western Blot | Expression of hemoglobin in IPF declined | (3) |
| Korfei et al. 2011 | 14 IPF  10 Control | Germany | 10  4 | 5  5 | 54.3±3.9 | 39.7±4.1 | Tissue | 2DE, MALDI-TOF, Western Blot, Immunohistochemistry | Kruskal Wallis test, Mann-Whitney U | 51 proteins UP-regulated and 38 proteins Down regulated in patients compared to the control groups | -------------- | --------- | Proteomic method are useful for detection the pathogenesis of IPF | (51) |
| Korfei et al. 2013 | 14 IPF  10 Control | Germany | 10  4 | 5  5 | 54.29±14.40 | 46.20±18.25 | Tissue, bronchoalveolar lavage fluid | 2DE, MALDI-TOF, Western Blot, Immunohistochemistry | Kruskal Wallis test | 14 proteins UP-regulation and 12 proteins Down regulated in patients compared to control the groups | -------------- | ---------- | Use of proteomic method help to distinguish different pathogenesis involved in IPF | (52) |
| Landi et al. 2014 | 7 IPF  10 Control | Italy | 6  1 | 5  5 | 63.5±2.3 | 65.3±8 | bronchoalveolar lavage fluid | 2DE, LC/MS-MS, MALDI-ToF–ToF/MS | Mann-Whitney U and Kruskal Wallis test, T test, Dunn’s test | 65 proteins were significantly different between the patients and controls | ------------ | Western Blot | Using proteomic and system biology methods help for diagnosis pathway involved in IPF | (53) |
| Landi et al. 2019 | 7 IPF-SSC  10 Control | Italy | 4  7 | 5  5 | 67.5 | 65.3 | bronchoalveolar lavage fluid | 2DE, MALDI-ToF/ToF | Mann-Whitney U and Kruskal Wallis test, T test, Dunn’s test | 21 proteins UP-regulated and 2 proteins Down regulated in patients compared to the control group | ------------ | Western Blot | Using proteomic method help to determine  the biomarkers and target therapy | (54) |
| Moodley et.al 2019 | 25 IPF  25 Control | Australia | 20  5 | 15  10 | 71.5 | 76 | Plasma | iTRAQ, HPLC, LC/MS-MS | Fisher’s exact test, Mann–Whitney U-test, Kruskal Wallis test, T test | One protein UP-regulation and 5 proteins Down regulation in patients compare to control groups | ------------ | Western Blot | Use of proteomic method help for pathway and molecular pathogenesis | (55) |
| Niu et al. 2017 | 60 IPF  60 Control | China | 40  20 | 36  24 | 72.5±10.56 | 67.98±8.48 | Plasma | Nano LC-MS-MS, iTRAQ, HPLC | ANOVA | 38 proteins UP-regulation and 59 proteins Down regulation in patients compare to control groups | Roc curve, Specificity, Sensitive | ELISA | Use of proteomic method help for detection pathogenesis and design target therapy | (56) |
| Dwyer et al. 2017 | 60 IPF  21 Control | USA | 41  19 | 15  6 | 64.56±7.74 | 69.29±10.12 | Plasma | SOMAscan, | Students t-test and Pearson χ2 squared test | 48 proteins UP-regulation and 116 proteins Down regulation in patients compare to control groups | -------------- | ---------- | Use of proteomic method help for detection pathogenesis | (57) |
| Ohlmeier et al. 2010 | 4 IPF  4 Control | Finland | ---- | ---- | ------ | ------ | bronchoalveolar lavage fluid, Tissue | 2DE, ELISA, | Mann–Whitney U | RAGE (FL-RAGE, cRAGE)  , ADAM10 and ADAM10 variant decreased in patients compare to Control group | ------------- | Western Blot | RAGE protein involved in pathogenesis of IPF | (45) |
| Rottoli et al.2005 | 13 IPF  5 Control | Italy | 10  3 | 3  2 | 60.32±9.35 | 42.16±13.51 | bronchoalveolar lavage fluid | ELISA, Western Blot, 2DE | Shapiro-Wilk’s test, T test | 9 proteins UP-regulation and 2 proteins Down regulation in patients compare to control groups | ------------- | ----------- | Detection Protein related oxidation help to diagnosis biology of IPF | (58) |
| Saraswat et al. 2020 | 17 IPF  19 Control | India | 14  3 | 14  5 | 71 | 73 | Plasma | UPLC-UDMS | T test, Mann–Whitney U, Partial least square-discriminant | 40 proteins UP-regulation and 122 proteins Down regulation in patients compare to control groups | R2, Q2, Roc curve, Specificity, Sensitive | ---------- | complement activation and oxidative damage involving in pathogenesis of IPF | (59) |
| Schiller et al. 2017 | 16 IPF  3 Control | Germany | 13  4 | 3  1 | 61.06±7.1 | 70.15±8.1 | Tissue | MS/MS, Wesrwen Blot | T test | 18 proteins UP-regulation and 25 proteins Down regulation in patients compare to control groups | ------------- | ----------- | Production antibody cause autoimmunity in IPF patients | (60) |
| Tian et al. 2019 | 20 IPF  20 Control | China | 19  1 | 17  3 | 63.86±10.58 | 63.67±8.28 | Lung Tissue | nano LC–MS/M, iTRAQ, LC–MS/MS, HPLC | T test, Mann–Whitney U | 455 proteins UP-regulation and 207 proteins Down regulation in patients compare to control groups | ----------- | Immunohistochemistry, Western Blot | Use of proteomic method help for detection pathogenesis and design target therapy | (61) |
| Todd et al. 2019 | 300 IPF  100 Control | USA | 223  77 | 74  26 | 70(65-75) | 66(63-71.5) | Plasma | SOMAscan | Pearson correlation, logistic regression, | 1305 proteins had significantly different levels between patients and controls | -------------- | ---------- | Use of proteomic method help for detection pathogenesis and design target therapy | (62) |
| Wattiez et al. 2000 | 3 IPF  5 Control | Belgium | 1  2 | 3  2 | 49(37-72) | 56(35-75) | bronchoalveolar lavage fluid | 2DE, Electroblotting | T test | 18 proteins UP-regulation and 3 proteins Down regulation in patients compare to control groups | -------------- | ---------- | Use of proteomic method help for detection pathogenesis | (63) |
| Stijn et al. 2013 | 11 IPF  10 Control | Belgium | 9  2 | 5  5 | 65(61-72) | 45(41-75) | bronchoalveolar lavage fluid | multiplex SearchLight | ANOVA, Kruskal-Wallis test, Mann–Whitney U test | 8 proteins UP-regulation and one protein Down regulation in patients compare to control groups | -------------- | ----------- | Use of proteomic method help for detection pathogenesis | (64) |
| Zhang et al.2018 | 20 IPF  20 Control | China | 14  6 | 14  6 | 61.57±4.03 | 59.09±4.13 | Serum | 2D-LC-MS/MS, iTRAQ | Kruskal-Wallis test, | 38 proteins UP-regulation and 59 proteins Down regulation in patients compare to control groups | --------------- | ELISA | Use of proteomic method help for detection pathogenesis | (65) |
| Ohlmeier et al. 2016 | 9 IPF  9 Control | Finland | 7  2 | 2  7 | 54.8±8.5 | 59.8±13.8 | Lung Tissue | 2DE, MALDI-TOF/T, OF, | Chi-Square, ANOVA, Kruskal-Wallis test, Mann-Whitney U-test | 2 proteins UP-regulation and 4 proteins Down regulation in patients compare to control groups | ------------- | ELISA, Western Blot | Evaluation of biomarker useful for detection pathogenesis of disease | (43) |
| Ahrman et al.2018 | 6 IPF  5 Control | Sweden | 1  5 | 4  1 | 56(50-65) | 55(39-65) | Lung Tissue | LC-MS/MS, SDS-PAGE, Immunohistochemistry | Kruskal-Wallis test | 7 proteins UP-regulation and 4 proteins Down regulation in patients compare to control groups | ---------------- | ----------- | Imbalance between protease-protease inhibitor resulting impairing tissue remodeling that involved in pathogenesis of IPF | (17) |
| Hara et al.2012 | 28 IPF  23 Control | Japan | 23  5 | 17  6 | 63.2 ± 10.2 | 28.0 ± 8.6 | bronchoalveolar lavage fluid, Serum | EISA, LCMS-IT-TOF, Immunohistochemistry, 2DE | KruskaleWallis  test, the post hoc Scheffe´ test, Pearson’s correlation  coefficient, log-rank test | S100A9 Up-regulation in patients group compare to Control group | Roc curve, Specificity, Sensitive | ---------- | Evaluation of S100A9 useful for differentiation IPF from Other disease | (66) |
| Bargagli et al. 2009 | 30 IPF  12 Control | Germany | 23  7 | 6  6 | 64.22±9.50 | 38.50±11.68 | bronchoalveolar lavage fluid | 2DE, Immunohistochemistry, ELISA | Chi-Square, , Kruskal-Wallis test, Mann-Whitney U-test, Spearman's rank correlation | MIF Upregulation in patients group compare to Control group | ----------- | --------- | MIF involved in pathogenesis of IPF | (67) |
| Landi et al. 2013 | 7 IPF  10 Control | Italy | 6  1 | 5  5 | 63.5±2.3 | 65.3±8.0 | bronchoalveolar lavage fluid | 2DE, MALDI-ToF/ToF-MS, LC–MS/MS | Kruskal-Wallis test, Mann-Whitney U-test, Dunn's test | 33 proteins UP-regulation and 18 proteins Down regulation in patients compare to control groups | ------------ | Western Blot | Some protein directly and indirectly involved in pathogenesis if IPF | (68) |

Abbreviation: IPF: Idiopathic Pulmonary Fibrosis, 2DE: Two-dimensional gel electrophoresis, LC/MS-MS: Liquid Chromatography with tandem mass spectrometry, LC-MRM: Liquid Chromatography Multiple reaction monitoring, MALDI-TOF: MALDI coupled to time-of-flight, MALDI-ToF–ToF/MS: MALDI coupled to time-of-flight mass spectrometry, iTRAQ: Isobaric tags for relative and absolute quantitation; HPLC: High-performance liquid chromatography, SDS-PAGE: sodium dodecyl sulphate–polyacrylamide gel electrophoresis, ELISA: Enzyme-linked immunosorbent assay, HPLC: High-performance liquid chromatography, LC-IMS-MS: Liquid Chromatography-Ion mobility spectrometry–mass spectrometry.

Table 3. Summary detail information of studies related to Asthma.

| **Author (year)** | **Sample size** | **Country** | **Male/Female** | | **Mean age ± SD** | | **Biological specimens** | **Technological platform used** | **Statistical analysis** | **Proteomic markers (UP/Down regulation)** | **Model characteristics** | **Validation** | **Final finding** | **Ref** |
| --- | --- | --- | --- | --- | --- | --- | --- | --- | --- | --- | --- | --- | --- | --- |
|  |  |  | **Case** | **Control** | **Case** | **Control** |  |  |  |  |  |  |  |  |
| Bhowmik et al. 2019 | 5 Asthma  5 Control | India | 2  3 | 3  2 | 62(34-76) | 34(26-44) | Plasma | SDS PAGE, ELISA, LC-MS/MS | T test | 2 proteins Down regulated in patients compared to the control group | ------------- | Western Blot | ApoE and IL-33 can be used as prognostic markers in Asthma patients | (2) |
|  | 5 COPD  5 Control |  | 5  0 | 3  2 | 62(55-70) | 34(26-44) |  |  |  |  |  |  |  |  |
| Cederfur et al. 2012 | 4 COPD  4 Control | Sweden | 3  1 | 2  2 | 28.75(24-31) | 28(26-31) | Bronchoalveolar lavage | LC-MS/MS, Immunohistochemistry, Affinity chromatography | -------- |  | ------------- | ----------- | The profile of galectin bound proteins was different between Asthma and heathy personals | (69) |
| Terracciano et al. 2011 | 8 Asthma  5 Control | Italy | 2  6 | 3  2 | 57 ± 13 | 55±21 | Sputum, Whole human saliva | MALDI-TOF/TOF MS, MALDI MS, | T test | 2 proteins Down-regulated in patients compared to the control group | ---------- | -------- | Use of proteomic methods can be useful for inflammation monitoring in Asthma by biomarkers | (39) |
| Gomes-Alves et al 2010 | 16 COPD  54 Control | Portugal | 12  4 | 21  33 | 63.2±5.8 | 41.6±14.5 | Serum | SELDI-TOF | Logistic regression, Kruskal-Wallis, T test, Dunn's Multiple Comparison Test, | 6 proteins differently expressed between two groups | Sensitivity, specificity, ROC curve | ------- | Evaluation of protein biomarkers can be useful for diagnosis and management of lung disease such as COPD | (35) |
| Verrills et al. 2011 | 21 Asthma  17 Control | Australia | 11  10 | 8  9 | 48.1± 12.7 | 44.2±14.4 | Platelet-depleted plasma | 2D-DIGE, MALDI-TOF/TOF | Logistic regression, T test, ANOVA, Spearman’s rank correlation | 9 proteins UP-regulated and 9 protein Down regulated in patients compared to the control group | Sensitivity, specificity, ROC curve | ELISA, Western Blot | Proteomic biomarkers showed that Iron metabolism and Acute phase response are involved in pathogenesis of air way in Asthma | (34) |
| Nicholas et al. 2010 | 11 Asthma  7 Control | UK | 5  6 | 4  3 | 49.6 (22–78) | 51.1 (35.5–69.3) | Sputum | 2DE, MS/MS, Immunohistochemistry, | t test, Mann-Whitney  U and Kendall’s tau b | 3 proteins UP regulated and one protein Down-regulated in patients compared to the control group | --------------- | Western blot , ELISA | Evaluation of protein expression can be useful to determine the pathogenesis of disease | (8) |
| Gharib et al. 2011 | 5 Asthmatic with EIB  5 Control | USA | 4  1 | 4  1 | 28.8(24-34) | 25.8 (18-40) | Sputum | LC-MS/MS, 2 DE | t Test, Wilcoxon | Seventeen proteins were significantly different between patients and controls | ------------ | Western Blot | Use of proteomic method is useful to determine the pathogenesis of Asthmatic and other airway diseases | (70) |
|  | 5 Asthmatic without EIB  5 Control |  | 4  1 | 4  1 | 31.4(23-54) | 25.8 (18-40) |  |  |  |  |  |  |  |  |
| Haggmark et al. 2015 | 17 Asthma  49 Control | Sweden | 6  11 | 26  23 | 22(18-52) | 24 (18-56) | Serum, bronchoalveolar lavage, Tissue | Immunohistochemistry, Microarray | Fisher’s exact test, median absolute deviation, | 2 proteins UP-regulated and 2 proteins Down regulated in patients compared to the control group | Roc curve, Specificity, Sensitive | ---------- | Different antigens were associated with variable concentration in patients | (71) |
| Ijpma et al. 2020 | 12 Asthma  19 Control | Canada | 7  5 | 11  8 | 39±4 | 43±3 | Tissue | uHPLC-MS/MS | logistic regression, t Test | 6 proteins UP-regulated and 21 proteins Down regulated in patients compared to the control group | ---------- | ----------- | Evaluation the proteins expression in asthma can be useful to design treatment | (72) |
| Jeong et al. 2007 | 6 Asthma  6 Control | China | 3  3 | 2  4 | 28 | 26 | CD3+ T-lymphocytes | 2D-PAGE, MADI-TOF-MS | MannWhitney U test, t Test | 13 proteins UP-regulated and 12 proteins Down regulated in patients compared to the control group | ---------------- | ----------- | Proteomic analysis is useful to determine the therapeutic targets in Asthmatic patients | (73) |
| Jiang et al. 2016 | 42 steroid sensitive asthma  35 Control | China | 20  22 | 18  17 | 45.8±11.6 | 39.5±17.1 | Serum | 2D-DIGE, MADI-TOF-MS, | t Test, ANOVA, Kruskal-Wallis test, Pearson correlation | 4 proteins UP-regulated and 3 proteins Down regulated in patients compared to the control group | Roc curve, Specificity, Sensitive | ELISA, Western Blot | Evaluation of serum VDBP may serve as a useful biomarker to predict steroid resistance in asthma patients | (74) |
|  | 36 steroid resistance asthma  35 Control |  | 17  19 | 18  17 | 43.5±18.4 | 39.5±17.1 |  |  |  |  |  |  |  |  |
| Kim et al. 2014 | 8 Asthma  8 AERD | South Korea | 4  4 | 3  5 | 52(32-64) | 47(33-65) | Plasma | HPLC, 2DE, MALDI-TOF-MS | Kruskal-Wallis H test, ManneWhitney | Apo H proteins UP-regulated in patients compared to the control group | Roc curve, Specificity, Sensitive | ELISA | Apo H may be involved in the pathogenesis of Asthma patients | (75) |
|  | 53 Asthma  24 Control |  | 27  26 | 6  18 | 51(28-84) | 54(31-79) |  |  |  |  |  |  |  |  |
| Larsen et al.2006 | 6 Asthma  3 Control | Sweden | ---- | ---- | ------ | ----- | Bronchoalveolar lavage fluid | 2DE, MALDI-TOF-MS, Western Blot | ManneWhitney | Haptoglobin proteins UP-regulated in patients compared tothe control group | ------------ | ---------- | Haptoglobin involved in airway remodeling in Asthma patients | (76) |
| Lasser et al. 2016 | 13 Asthma  14 Control | Sweden | 3  10 | 5  9 | 44±3 | 40±3 | Nasal lavage fluid, exosome | Strong cation exchange (SCX) chromatography, NanoLC‑MS/MS, Tandem mass tags labelling of peptide, LC–MS/MS | Mann– Whitney U test, Kruskal–Wallis test | 9 proteins UP-regulated and 12 proteins Down regulated in Asthma compared to the control group | -------------- | Western Blot | Decreased antimicrobial proteins contributed to infection in patients and cause disease progression | (77) |
|  | 15 asthma and chronic rhinosinusitis  14 Control |  | 4  11 | 5  9 | 38±3 | 40±3 |  |  |  |  |  |  |  |  |
| Fontarigo et al.2020 | 49 moderate-severe allergic asthma  32 Control | Spain | 23  26 | 15  17 | 39(18-68) | 43(22-61) | Serum | nanoLC/MS-MS, iTRAQ, HPLC | Kruskal–Wallis one-way, t Test, Dunn's test | Eighteen proteins were significantly different between patient and control group | Roc curve, Specificity, Sensitive | ELISA | Use of proteomic method is useful for detecting biomarkers for diagnosis, prognosis and target therapy in Asthma patients | (78) |
|  | 53 intermittent-mild allergic asthma  32 Control |  | 25  28 | 15  17 | 36(20-66) | 43(22-61) |  |  |  |  |  |  |  |  |
|  | 43 moderate-severe non-allergic asthma  32 Control |  | 13  30 | 15  17 | 54(24-68) | 43(22-61) |  |  |  |  |  |  |  |  |
|  | 47 intermittent-mild non-allergic asthma  32 Control |  | 8  39 | 15  17 | 52(29-72) | 43(22-61) |  |  |  |  |  |  |  |  |
|  | 43 allergic rhinitis  32 Control |  | 24  19 | 15  17 | 35(18-55) | 43(22-61) |  |  |  |  |  |  |  |  |
| O’Neil et al.2011 | 12 Asthma  3 Control | Sweden | 2  10 | 0  3 | 57(38-67) | 55(42-67) | Tissue | nanoLC-MS/MS, iTRAQ, HPLC | t Test | 7 proteins Down regulated in patient compare to control groups | -------------- | ---------- | Quantitative proteomics is useful to determine disease pathogenesis | (79) |
| Suojalehto et al.2015 | 40 Asthma and allergic  rhinitis  42 Control | USA | 21  19 | 17  25 | 32.5±1 | 33.5±1.8 | Sputum and nasal lavage fluid | 2D-DIGE, LC-S/MS, nanoLC-MS/MS, Q-TOF | Chi-square test, t Test, Mann-Whitney U-test, ANOVA, Kruskal-Wallis | 2 proteins UP-regulated and 10 proteins Down regulated in patientsd compare to the control group | Roc curve, Specificity, Sensitive | ELISA, Western Blot | FABP5 contribute to the airway remodeling and inflammation in Asthma by VEGF production. | (80) |
| Suojalehto et al.2018 | 8 Asthma related to protein allergen  8 Control | Finlad | 10  19 | 1  7 | 35.6±12.3 | 43.8±2.1 | nasal brush | 2D-DIGE, LC-S/MS, | Mann -Whitney U –test, Chi - square test, Spearman´s correlation, t Test | 77 proteins were significantly different between patient and control group | ----------- | Western Blot | Revealed biological activities of the protein expression changes are associated with allergic inflammation and asthma. | (81) |
|  | 8 Asthma related to isocyanate allergen  8 Control |  | 9  3 | 6  2 | 41.7±11.4 | 43.8±2.1 |  |  |  |  |  |  |  |  |
|  | 8 Asthma related to welding allergen  8 Control |  | 12  2 | 6  2 | 43.3±10.4 | 43.8±2.1 |  |  |  |  |  |  |  |  |
| Gupta et al.2012 | 20 Asthma  20 Control | India | 11  9 | ----- | 31.6±6.67 | 18-60 | Peripheral blood (erythrocyte) | SDS-PAGE | T-test | 6 proteins UP-regulated and 2 proteins Down regulated in patients compared to the control | ------------ | -------- | Alter proteins expression causes clinical symptoms, and is involved in the pathogenesis | (82) |
| Wu et al. 2005 | 4 Asthma  3 Control | Massachusetts | 1  3 | 1  2  33.25 | ------ | 34 | BALF | HPLC, SDS-PAGE, nano-LC-/MS/MS, | T-test, | 441 proteins were significantly different between patient and control group | ------------- | ELISA | Use of proteomic method can help to determine the pathogenesis of Asthma | (83) |
| Gray et al.2008 | 24 Asthma  20 Control | UK | 8  15 | 9  11 | 47.8 ± 2.2 | 36.4 ± 2.1 | Sputum | SELDI-TOF, ELISA | Mann-Whitney, T test | 105 proteins were significantly different between patient and control group | ------------- | -------- | Use of proteomic methods can be useful for detecting biomarkers related to inflammation in early stage of lung disease | (38) |
| Takahashi et al.2018 | 11 severe Asthmas with current smoker  18 Control | UK | 6  5 | 6  12 | 50.0±10.6 | 39.9±13.8 | bronchial brushings, biopsies and sputum | Microarray | Fisher’s exact test, Kruskal-Wallis test, ANOVA, | 118 proteins were significantly different between patient and control group | ------------ | ------------ | Evaluation of proteins expression help distinguish different clinical of Asthma | (84) |
|  | 22 severe  Asthma with Ex-smoker  18 Control |  | 8  14 | 6  12 | 55.7±9.7 | 39.9±13.8 |  |  |  |  |  |  |  |  |
|  | 37 Severe asthma with non-smoker  18 Control |  | 15  22 | 6  12 | 52.6±13.3 | 39.9±13.8 |  |  |  |  |  |  |  |  |
| Tariq et al. 2019 | 311 Severe asthma non smoker  101 Control | UK | 106  205 | 62  39 | 53(43-62) | 34(27-49) | Sputum | LC-IMS-MS | Shapiro-Wilk normality test, Mann–Whitney U test, multiple logistic regression | one protein UP-regulated and 11 proteins Down regulated in patients compared to the control group | ------------ | ---------- | Evaluation of proteins expression help to distinguish different phenotypes of Asthma | (85) |
|  | 110 Severe asthma with smoker  101 Control |  | 54  56 | 62  39 | 55(48-61) | 34(27-49) |  |  |  |  |  |  |  |  |
|  | 88 Mild/Moderate asthma  101 Control |  | 44  44 | 62  39 | 42.5(28-52.8) | 34(27-49) |  |  |  |  |  |  |  |  |

Abbreviation: 2DE: Two-dimensional gel electrophoresis, LC/MS-MS: Liquid Chromatography with tandem mass spectrometry, LC-MRM: Liquid Chromatography Multiple reaction monitoring, MALDI-TOF: MALDI coupled to time-of-flight, MALDI-ToF–ToF/MS: MALDI coupled to time-of-flight mass spectrometry, iTRAQ: Isobaric tags for relative and absolute quantitation; HPLC: High-performance liquid chromatography, SDS-PAGE: sodium dodecyl sulphate–polyacrylamide gel electrophoresis, ELISA: Enzyme-linked immunosorbent assay, HPLC: High-performance liquid chromatography, LC-IMS-MS: Liquid Chromatography-Ion mobility spectrometry–mass spectrometry

Table 4. Summary detail information of studies related to BO and mustard gas expose victims.

| **Author (year)** | **Sample size** | **Country** | **Male/Female** | | **Mean age ± SD** | | **Biological specimens** | **Technological platform used** | **Statistical analysis** | **Proteomic markers (UP/Down regulation)** | **Model characteristics** | **Validation** | **Final finding** | **Ref** |
| --- | --- | --- | --- | --- | --- | --- | --- | --- | --- | --- | --- | --- | --- | --- |
|  |  |  | **Case** | **Control** | **Case** | **Control** |  |  |  |  |  |  |  |  |
| Nelsestuen et al.2005 | 56 Bronchiolitis obliterans  47 Control | USA | ----- | ----- | ---- | ---- | Broncho alveolar lavage fluid | MALDI-TOF, HPLC | NA | Human neutrophil peptides 1–3 UP-regulated Bronchiolitis obliterans compared to the control group. | Roc Curve , Specify, Sensitivity, | ELISA | Elevated HNP levels are associated  with the onset of BOS | (86) |
| Zhang et al. 2006 | 30 Bronchiolitis obliterans  27 Control | USA | ----- | ----- | ---- | ---- | Broncho alveolar lavage fluid | MALDI-TOF | T test | 2 proteins UP-regulated in Bronchiolitis obliterans compared to the control group | Roc Curve ,Specify, Sensitivity, | ---------- | Use of proteomic can be useful for detection of biomarkers for disease monitoring | (87) |
| Shahriary et al.2015 | 10 Mustard-expose  10 Control | Iran | 10  0 | 10  0 | 52.5±3.62 | 54.4±2.9 | Blood neutrophil | 2D -SDS PAGE, MALDI-TOF-MS/MS | ANOVA, Tukey’s range test, post-hoc  analysis | 7 proteins UP regulated and 6 proteins Down regulated in patients compared to the control group | -------------------- | --------------- | Apart from inflammation and oxidative stress, imbalance of protease and anti-protease involved in the pathogenesis of mustard expose patients. | (15) |
| Majd et al. 2020 | 10 SM exposed  without pulmonary complications  10 Control | Iran | 7  3 | 8  2 | 50.6 ± 9.3 | 59.1 ± 12.4 | Serum | 2DE | ------------------ | 14 proteins UP-regulated, while 8 proteins Down regulated in patients compare to the control group | ----------------------- | ----------------- | Evaluation of protein biomarkers is useful for treatment and understanding the pathogenesis of disease | (88) |
|  | 10 SM exposed  with pulmonary complications  10 Control |  | 10  0 | 8  2 | 48.9 ± 9.8 | 59.1 ± 12.4 |  |  |  |  |  |  |  |  |
| Pajoohesh et al.2017 | 8 Mustard-expose  8 Control | Iran | 8  0 | 8  0 | 48.0±6.7 | 48.0±6.7 | Corneal epithelium | SDS-PAGE, MALDI-TOF-MS/MS | ANOVA, Progenesis SameSpots software | 6 proteins UP-regulated, while 18 proteins Down regulated in patients compared to the control group | ------------------ | Western blot | Detection of protein biomarkers can be useful for involved pathways in apoptosis and cell death of patients. | (89) |
| Pashandi et al. 2015 | 10 Mustard-expose  10 Control | Iran | 10  0 | 10  0 | 54.0±4.2 | 54.0±4.2 | Serum | 2DE, MALDI-TOF-MS/MS | ANOVA, Student’s t-test | 6 proteins UP-regulated and 2 proteins Down-regulated in patients compared to the control group | ---------------- | Western blot | Change in serum proteins leads to exacerbate ocular injury | (90) |
| Mehrani et al. 2011 | 20 Mustard-expose  20 Control | Iran | 20  0 | 20  0 | 43.6±2.8 | 40.1±3.6 | Plasma | SDS-PAGE, MALDI-TOF-MS/MS | Unpaired Student’s t-test | 2 proteins UP-regulated in patients compared to the control group | --------------- | ------------ | Result showed that tissue remodeling involved in pathogenesis of lung patients | (91) |
| Mehrani et al. 2009 | 10 Mustard-expose (Mild)  10 Control | Iran | 10  0 | 10  0 | 40.3±2.6 | 36.8±3.9 | Broncho alveolar lavage | 2DE-SDS PAGE, MALDI-TOF-TOF-MS | Post hoc Dunnett’s test | 5 proteins UP-regulated, while 5 proteins Down-regulated in patients compare to the control group | ---------------- | -------------- | The proteomic method is useful for detection and targeting biomarkers for the treatment of mustard-expose patients | (92) |
|  | 10 Mustard-expose(Moderate)  10 Control |  | 10  0 | 10  0 | 43.0±1.8 | 36.8±3.9 |  |  |  |  |  |  |  |  |
|  | 10 Mustard-expose(severe)  10 Control |  | 10  0 | 10  0 | 42.0±2.8 | 36.8±3.9 |  |  |  |  |  |  |  |  |

Abbreviation: 2DE: Two-dimensional gel electrophoresis, LC/MS-MS: Liquid Chromatography with tandem mass spectrometry, LC-MRM: Liquid Chromatography Multiple reaction monitoring, MALDI-TOF: MALDI coupled to time-of-flight, MALDI-ToF–ToF/MS: MALDI coupled to time-of-flight mass spectrometry, iTRAQ: Isobaric tags for relative and absolute quantitation; HPLC: High-performance liquid chromatography, SDS-PAGE: sodium dodecyl sulphate–polyacrylamide gel electrophoresis, ELISA: Enzyme-linked immunosorbent assay, HPLC: High-performance liquid chromatography.

1. Kononikhin AS, Fedorchenko KY, Ryabokon AM, Starodubtseva NL, Popov IA, Zavialova MG, et al. [Proteomic analysis of exhaled breath condensate for diagnosis of pathologies of the respiratory system]. Biomeditsinskaia khimiia. 2015;61(6):777-80.

2. Bhowmik M, Majumdar S, Dasgupta A, Gupta Bhattacharya S, Saha S. Pilot-Scale Study Of Human Plasma Proteomics Identifies ApoE And IL33 As Markers In Atopic Asthma. Journal of asthma and allergy. 2019;12:273-83.

3. Ishikawa N, Ohlmeier S, Salmenkivi K, Myllärniemi M, Rahman I, Mazur W, et al. Hemoglobin α and β are ubiquitous in the human lung, decline in idiopathic pulmonary fibrosis but not in COPD. Respiratory research. 2010;11(1):123.

4. Loi ALT, Hoonhorst S, van Aalst C, Langereis J, Kamp V, Sluis-Eising S, et al. Proteomic profiling of peripheral blood neutrophils identifies two inflammatory phenotypes in stable COPD patients. Respiratory research. 2017;18(1):100.

5. Baralla A, Fois AG, Sotgiu E, Zinellu E, Mangoni AA, Sotgia S, et al. Plasma Proteomic Signatures in Early Chronic Obstructive Pulmonary Disease. Proteomics Clinical applications. 2018;12(3):e1700088.

6. Linja-aho A, Mazur W, Toljamo T, Nieminen P, Ohlmeier S, Rönty M, et al. Distribution and levels of alpha-1-antitrypsin in the lung and plasma in smokers and chronic obstructive pulmonary disease. APMIS : acta pathologica, microbiologica, et immunologica Scandinavica. 2013;121(1):11-21.

7. Casado B, Iadarola P, Pannell LK, Luisetti M, Corsico A, Ansaldo E, et al. Protein expression in sputum of smokers and chronic obstructive pulmonary disease patients: a pilot study by CapLC-ESI-Q-TOF. Journal of proteome research. 2007;6(12):4615-23.

8. Nicholas BL, Skipp P, Barton S, Singh D, Bagmane D, Mould R, et al. Identification of lipocalin and apolipoprotein A1 as biomarkers of chronic obstructive pulmonary disease. American journal of respiratory and critical care medicine. 2010;181(10):1049-60.

9. Titz B, Sewer A, Schneider T, Elamin A, Martin F, Dijon S, et al. Alterations in the sputum proteome and transcriptome in smokers and early-stage COPD subjects. Journal of proteomics. 2015;128:306-20.

10. Alexandre BM, Charro N, Blonder J, Lopes C, Azevedo P, Bugalho de Almeida A, et al. Profiling the erythrocyte membrane proteome isolated from patients diagnosed with chronic obstructive pulmonary disease. Journal of proteomics. 2012;76 Spec No.:259-69.

11. Sun L, Yin H, Liu M, Xu G, Zhou X, Ge P, et al. Impaired albumin function: a novel potential indicator for liver function damage? Annals of medicine. 2019;51(7-8):333-44.

12. Tu C, Mammen MJ, Li J, Shen X, Jiang X, Hu Q, et al. Large-scale, ion-current-based proteomics investigation of bronchoalveolar lavage fluid in chronic obstructive pulmonary disease patients. Journal of proteome research. 2014;13(2):627-39.

13. Brandsma CA, Guryev V, Timens W, Ciconelle A, Postma DS, Bischoff R, et al. Integrated proteogenomic approach identifying a protein signature of COPD and a new splice variant of SORBS1. Thorax. 2020;75(2):180-3.

14. Merkel D, Rist W, Seither P, Weith A, Lenter MC. Proteomic study of human bronchoalveolar lavage fluids from smokers with chronic obstructive pulmonary disease by combining surface-enhanced laser desorption/ionization-mass spectrometry profiling with mass spectrometric protein identification. Proteomics. 2005;5(11):2972-80.

15. Shahriary A, Mehrani H, Ghanei M, Parvin S. Comparative proteome analysis of peripheral neutrophils from sulfur mustard-exposed and COPD patients. Journal of immunotoxicology. 2015;12(2):132-9.

16. Tan DBA, Ito J, Peters K, Livk A, Lipscombe RJ, Casey TM, et al. Protein Network Analysis Identifies Changes in the Level of Proteins Involved in Platelet Degranulation, Proteolysis and Cholesterol Metabolism Pathways in AECOPD Patients. Copd. 2020;17(1):29-33.

17. Åhrman E, Hallgren O, Malmström L, Hedström U, Malmström A, Bjermer L, et al. Quantitative proteomic characterization of the lung extracellular matrix in chronic obstructive pulmonary disease and idiopathic pulmonary fibrosis. Journal of proteomics. 2018;189:23-33.

18. Lee EJ, In KH, Kim JH, Lee SY, Shin C, Shim JJ, et al. Proteomic analysis in lung tissue of smokers and COPD patients. Chest. 2009;135(2):344-52.

19. Li F, Xu D, Wang J, Jing J, Li Z, Jin X. Comparative proteomics analysis of patients with quick development and slow development Chronic Obstructive Pulmonary Disease (COPD). Life sciences. 2020;256:117829.

20. Chen H, Song Z, Qian M, Bai C, Wang X. Selection of disease-specific biomarkers by integrating inflammatory mediators with clinical informatics in AECOPD patients: a preliminary study. Journal of cellular and molecular medicine. 2012;16(6):1286-97.

21. Chen H, Wang Y, Bai C, Wang X. Alterations of plasma inflammatory biomarkers in the healthy and chronic obstructive pulmonary disease patients with or without acute exacerbation. Journal of proteomics. 2012;75(10):2835-43.

22. Baraniuk JN, Casado B, Pannell LK, McGarvey PB, Boschetto P, Luisetti M, et al. Protein networks in induced sputum from smokers and COPD patients. International journal of chronic obstructive pulmonary disease. 2015;10:1957-75.

23. Gao J, Ohlmeier S, Nieminen P, Toljamo T, Tiitinen S, Kanerva T, et al. Elevated sputum BPIFB1 levels in smokers with chronic obstructive pulmonary disease: a longitudinal study. American journal of physiology Lung cellular and molecular physiology. 2015;309(1):L17-26.

24. Bandow JE, Baker JD, Berth M, Painter C, Sepulveda OJ, Clark KA, et al. Improved image analysis workflow for 2-D gels enables large-scale 2-D gel-based proteomics studies--COPD biomarker discovery study. Proteomics. 2008;8(15):3030-41.

25. Gao J, Törölä T, Li CX, Ohlmeier S, Toljamo T, Nieminen P, et al. Sputum Vitamin D Binding Protein (VDBP) GC1S/1S Genotype Predicts Airway Obstruction: A Prospective Study in Smokers with COPD. International journal of chronic obstructive pulmonary disease. 2020;15:1049-59.

26. López-Sánchez LM, Jurado-Gámez B, Feu-Collado N, Valverde A, Cañas A, Fernández-Rueda JL, et al. Exhaled breath condensate biomarkers for the early diagnosis of lung cancer using proteomics. American journal of physiology Lung cellular and molecular physiology. 2017;313(4):L664-l76.

27. Franciosi L, Postma DS, van den Berge M, Govorukhina N, Horvatovich PL, Fusetti F, et al. Susceptibility to COPD: differential proteomic profiling after acute smoking. PloS one. 2014;9(7):e102037.

28. Franciosi L, Govorukhina N, Fusetti F, Poolman B, Lodewijk ME, Timens W, et al. Proteomic analysis of human epithelial lining fluid by microfluidics-based nanoLC-MS/MS: a feasibility study. Electrophoresis. 2013;34(18):2683-94.

29. Pastor MD, Nogal A, Molina-Pinelo S, Meléndez R, Salinas A, González De la Peña M, et al. Identification of proteomic signatures associated with lung cancer and COPD. Journal of proteomics. 2013;89:227-37.

30. Saleem M, Raza SK, S GM. A comparative protein analysis of lung cancer, along with three controls using a multidimensional proteomic approach. Experimental biology and medicine (Maywood, NJ). 2019;244(1):36-41.

31. Fumagalli M, Ferrari F, Luisetti M, Stolk J, Hiemstra PS, Capuano D, et al. Profiling the proteome of exhaled breath condensate in healthy smokers and COPD patients by LC-MS/MS. International journal of molecular sciences. 2012;13(11):13894-910.

32. Kohler M, Sandberg A, Kjellqvist S, Thomas A, Karimi R, Nyrén S, et al. Gender differences in the bronchoalveolar lavage cell proteome of patients with chronic obstructive pulmonary disease. The Journal of allergy and clinical immunology. 2013;131(3):743-51.

33. Yang M, Kohler M, Heyder T, Forsslund H, Garberg HK, Karimi R, et al. Long-term smoking alters abundance of over half of the proteome in bronchoalveolar lavage cell in smokers with normal spirometry, with effects on molecular pathways associated with COPD. Respiratory research. 2018;19(1):40.

34. Verrills NM, Irwin JA, He XY, Wood LG, Powell H, Simpson JL, et al. Identification of novel diagnostic biomarkers for asthma and chronic obstructive pulmonary disease. American journal of respiratory and critical care medicine. 2011;183(12):1633-43.

35. Gomes-Alves P, Imrie M, Gray RD, Nogueira P, Ciordia S, Pacheco P, et al. SELDI-TOF biomarker signatures for cystic fibrosis, asthma and chronic obstructive pulmonary disease. Clinical biochemistry. 2010;43(1-2):168-77.

36. Langereis JD, Schweizer RC, Lammers JW, Koenderman L, Ulfman LH. A unique protein profile of peripheral neutrophils from COPD patients does not reflect cytokine-induced protein profiles of neutrophils in vitro. BMC pulmonary medicine. 2011;11:44.

37. Sun P, Ye R, Wang C, Bai S, Zhao L. Identification of proteomic signatures associated with COPD frequent exacerbators. Life sciences. 2019;230:1-9.

38. Gray RD, MacGregor G, Noble D, Imrie M, Dewar M, Boyd AC, et al. Sputum proteomics in inflammatory and suppurative respiratory diseases. American journal of respiratory and critical care medicine. 2008;178(5):444-52.

39. Terracciano R, Preianò M, Palladino GP, Carpagnano GE, Barbaro MP, Pelaia G, et al. Peptidome profiling of induced sputum by mesoporous silica beads and MALDI-TOF MS for non-invasive biomarker discovery of chronic inflammatory lung diseases. Proteomics. 2011;11(16):3402-14.

40. Hu R, Ouyang Q, Dai A, Tan S, Xiao Z, Tang C. Heat shock protein 27 and cyclophilin A associate with the pathogenesis of COPD. Respirology. 2011;16(6):983-93.

41. Merali S, Barrero CA, Bowler RP, Chen DE, Criner G, Braverman A, et al. Analysis of the plasma proteome in COPD: Novel low abundance proteins reflect the severity of lung remodeling. Copd. 2014;11(2):177-89.

42. Ohlmeier S, Vuolanto M, Toljamo T, Vuopala K, Salmenkivi K, Myllärniemi M, et al. Proteomics of human lung tissue identifies surfactant protein A as a marker of chronic obstructive pulmonary disease. Journal of proteome research. 2008;7(12):5125-32.

43. Ohlmeier S, Nieminen P, Gao J, Kanerva T, Rönty M, Toljamo T, et al. Lung tissue proteomics identifies elevated transglutaminase 2 levels in stable chronic obstructive pulmonary disease. American journal of physiology Lung cellular and molecular physiology. 2016;310(11):L1155-65.

44. Ohlmeier S, Mazur W, Linja-Aho A, Louhelainen N, Rönty M, Toljamo T, et al. Sputum proteomics identifies elevated PIGR levels in smokers and mild-to-moderate COPD. Journal of proteome research. 2012;11(2):599-608.

45. Ohlmeier S, Mazur W, Salmenkivi K, Myllärniemi M, Bergmann U, Kinnula VL. Proteomic studies on receptor for advanced glycation end product variants in idiopathic pulmonary fibrosis and chronic obstructive pulmonary disease. Proteomics Clinical applications. 2010;4(1):97-105.

46. York TP, van den Oord EJ, Langston TB, Edmiston JS, McKinney W, Webb BT, et al. High-resolution mass spectrometry proteomics for the identification of candidate plasma protein biomarkers for chronic obstructive pulmonary disease. Biomarkers : biochemical indicators of exposure, response, and susceptibility to chemicals. 2010;15(4):367-77.

47. Diao WQ, Shen N, Du YP, Liu BB, Sun XY, Xu M, et al. Fetuin-B (FETUB): a Plasma Biomarker Candidate Related to the Severity of Lung Function in COPD. Scientific reports. 2016;6:30045.

48. Liu Y, Liu H, Li C, Ma C, Ge W. Proteome Profiling of Lung Tissues in Chronic Obstructive Pulmonary Disease (COPD): Platelet and Macrophage Dysfunction Contribute to the Pathogenesis of COPD. International journal of chronic obstructive pulmonary disease. 2020;15:973-80.

49. Zhang X, Zhang J, Li Q, Wu X, Wang T, Wang Y. SELDI-TOF-MS in chronic obstructive pulmonary disease. Chin Sci Bull. 2013;58:634-40.

50. Foster MW, Morrison LD, Todd JL, Snyder LD, Thompson JW, Soderblom EJ, et al. Quantitative proteomics of bronchoalveolar lavage fluid in idiopathic pulmonary fibrosis. Journal of proteome research. 2015;14(2):1238-49.

51. Korfei M, Schmitt S, Ruppert C, Henneke I, Markart P, Loeh B, et al. Comparative proteomic analysis of lung tissue from patients with idiopathic pulmonary fibrosis (IPF) and lung transplant donor lungs. Journal of proteome research. 2011;10(5):2185-205.

52. Korfei M, von der Beck D, Henneke I, Markart P, Ruppert C, Mahavadi P, et al. Comparative proteome analysis of lung tissue from patients with idiopathic pulmonary fibrosis (IPF), non-specific interstitial pneumonia (NSIP) and organ donors. Journal of proteomics. 2013;85:109-28.

53. Landi C, Bargagli E, Carleo A, Bianchi L, Gagliardi A, Prasse A, et al. A system biology study of BALF from patients affected by idiopathic pulmonary fibrosis (IPF) and healthy controls. Proteomics Clinical applications. 2014;8(11-12):932-50.

54. Landi C, Bargagli E, Carleo A, Refini RM, Bennett D, Bianchi L, et al. Bronchoalveolar lavage proteomic analysis in pulmonary fibrosis associated with systemic sclerosis: S100A6 and 14-3-3ε as potential biomarkers. Rheumatology (Oxford, England). 2019;58(1):165-78.

55. Moodley YP, Corte TJ, Oliver BG, Glaspole IN, Livk A, Ito J, et al. Analysis by proteomics reveals unique circulatory proteins in idiopathic pulmonary fibrosis. Respirology (Carlton, Vic). 2019;24(11):1111-4.

56. Niu R, Liu Y, Zhang Y, Zhang Y, Wang H, Wang Y, et al. iTRAQ-Based Proteomics Reveals Novel Biomarkers for Idiopathic Pulmonary Fibrosis. PloS one. 2017;12(1):e0170741.

57. O'Dwyer DN, Norman KC, Xia M, Huang Y, Gurczynski SJ, Ashley SL, et al. The peripheral blood proteome signature of idiopathic pulmonary fibrosis is distinct from normal and is associated with novel immunological processes. Scientific reports. 2017;7:46560.

58. Rottoli P, Magi B, Cianti R, Bargagli E, Vagaggini C, Nikiforakis N, et al. Carbonylated proteins in bronchoalveolar lavage of patients with sarcoidosis, pulmonary fibrosis associated with systemic sclerosis and idiopathic pulmonary fibrosis. Proteomics. 2005;5(10):2612-8.

59. Saraswat M, Joenväärä S, Tohmola T, Sutinen E, Vartiainen V, Koli K, et al. Label-free plasma proteomics identifies haptoglobin-related protein as candidate marker of idiopathic pulmonary fibrosis and dysregulation of complement and oxidative pathways. Scientific reports. 2020;10(1):7787.

60. Schiller HB, Mayr CH, Leuschner G, Strunz M, Staab-Weijnitz C, Preisendörfer S, et al. Deep Proteome Profiling Reveals Common Prevalence of MZB1-Positive Plasma B Cells in Human Lung and Skin Fibrosis. American journal of respiratory and critical care medicine. 2017;196(10):1298-310.

61. Tian Y, Li H, Gao Y, Liu C, Qiu T, Wu H, et al. Quantitative proteomic characterization of lung tissue in idiopathic pulmonary fibrosis. Clinical proteomics. 2019;16:6.

62. Todd JL, Neely ML, Overton R, Durham K, Gulati M, Huang H, et al. Peripheral blood proteomic profiling of idiopathic pulmonary fibrosis biomarkers in the multicentre IPF-PRO Registry. Respiratory research. 2019;20(1):227.

63. Wattiez R, Hermans C, Cruyt C, Bernard A, Falmagne P. Human bronchoalveolar lavage fluid protein two-dimensional database: study of interstitial lung diseases. Electrophoresis. 2000;21(13):2703-12.

64. Willems S, Verleden SE, Vanaudenaerde BM, Wynants M, Dooms C, Yserbyt J, et al. Multiplex protein profiling of bronchoalveolar lavage in idiopathic pulmonary fibrosis and hypersensitivity pneumonitis. Annals of thoracic medicine. 2013;8(1):38-45.

65. Zhang Y, Xin Q, Wu Z, Wang C, Wang Y, Wu Q, et al. Application of Isobaric Tags for Relative and Absolute Quantification (iTRAQ) Coupled with Two-Dimensional Liquid Chromatography/Tandem Mass Spectrometry in Quantitative Proteomic Analysis for Discovery of Serum Biomarkers for Idiopathic Pulmonary Fibrosis. Medical science monitor : international medical journal of experimental and clinical research. 2018;24:4146-53.

66. Hara A, Sakamoto N, Ishimatsu Y, Kakugawa T, Nakashima S, Hara S, et al. S100A9 in BALF is a candidate biomarker of idiopathic pulmonary fibrosis. Respiratory medicine. 2012;106(4):571-80.

67. Bargagli E, Olivieri C, Nikiforakis N, Cintorino M, Magi B, Perari MG, et al. Analysis of macrophage migration inhibitory factor (MIF) in patients with idiopathic pulmonary fibrosis. Respiratory physiology & neurobiology. 2009;167(3):261-7.

68. Landi C, Bargagli E, Bianchi L, Gagliardi A, Carleo A, Bennett D, et al. Towards a functional proteomics approach to the comprehension of idiopathic pulmonary fibrosis, sarcoidosis, systemic sclerosis and pulmonary Langerhans cell histiocytosis. Journal of proteomics. 2013;83:60-75.

69. Cederfur C, Malmström J, Nihlberg K, Block M, Breimer ME, Bjermer L, et al. Glycoproteomic identification of galectin-3 and -8 ligands in bronchoalveolar lavage of mild asthmatics and healthy subjects. Biochimica et biophysica acta. 2012;1820(9):1429-36.

70. Gharib SA, Nguyen EV, Lai Y, Plampin JD, Goodlett DR, Hallstrand TS. Induced sputum proteome in healthy subjects and asthmatic patients. The Journal of allergy and clinical immunology. 2011;128(6):1176-84.e6.

71. Häggmark A, Hamsten C, Wiklundh E, Lindskog C, Mattsson C, Andersson E, et al. Proteomic profiling reveals autoimmune targets in sarcoidosis. American journal of respiratory and critical care medicine. 2015;191(5):574-83.

72. Ijpma G, Kachmar L, Panariti A, Matusovsky OS, Torgerson D, Benedetti A, et al. Intrapulmonary airway smooth muscle is hyperreactive with a distinct proteome in asthma. The European respiratory journal. 2020;56(1).

73. Jeong HC, Lee SY, Lee EJ, Jung KH, Kang EH, Lee SY, et al. Proteomic analysis of peripheral T-lymphocytes in patients with asthma. Chest. 2007;132(2):489-96.

74. Jiang H, Chi X, Zhang X, Wang J. Increased serum VDBP as a risk predictor for steroid resistance in asthma patients. Respiratory medicine. 2016;114:111-6.

75. Kim HJ, Park JS, Heo JS, Moon KY, Park CS. Plasma apolipoprotein H levels are different between aspirin induced respiratory diseases and aspirin tolerant asthma. Pulmonary pharmacology & therapeutics. 2014;27(2):184-9.

76. Larsen K, Macleod D, Nihlberg K, Gürcan E, Bjermer L, Marko-Varga G, et al. Specific haptoglobin expression in bronchoalveolar lavage during differentiation of circulating fibroblast progenitor cells in mild asthma. Journal of proteome research. 2006;5(6):1479-83.

77. Lässer C, O'Neil SE, Shelke GV, Sihlbom C, Hansson SF, Gho YS, et al. Exosomes in the nose induce immune cell trafficking and harbour an altered protein cargo in chronic airway inflammation. Journal of translational medicine. 2016;14(1):181.

78. Nieto-Fontarigo JJ, González-Barcala FJ, Andrade-Bulos LJ, San-José ME, Cruz MJ, Valdés-Cuadrado L, et al. iTRAQ-based proteomic analysis reveals potential serum biomarkers of allergic and nonallergic asthma. Allergy. 2020;75(12):3171-83.

79. O'Neil SE, Sitkauskiene B, Babusyte A, Krisiukeniene A, Stravinskaite-Bieksiene K, Sakalauskas R, et al. Network analysis of quantitative proteomics on asthmatic bronchi: effects of inhaled glucocorticoid treatment. Respiratory research. 2011;12(1):124.

80. Suojalehto H, Kinaret P, Kilpeläinen M, Toskala E, Ahonen N, Wolff H, et al. Level of Fatty Acid Binding Protein 5 (FABP5) Is Increased in Sputum of Allergic Asthmatics and Links to Airway Remodeling and Inflammation. PloS one. 2015;10(5):e0127003.

81. Suojalehto H, Lindström I, Wolff H, Puustinen A. Nasal protein profiles in work-related asthma caused by different exposures. Allergy. 2018;73(3):653-63.

82. Gupta P, Vijayan VK, Bansal SK. Changes in protein profile of erythrocyte membrane in bronchial asthma. The Journal of asthma : official journal of the Association for the Care of Asthma. 2012;49(2):129-33.

83. Wu J, Kobayashi M, Sousa EA, Liu W, Cai J, Goldman SJ, et al. Differential proteomic analysis of bronchoalveolar lavage fluid in asthmatics following segmental antigen challenge. Molecular & cellular proteomics : MCP. 2005;4(9):1251-64.

84. Takahashi K, Pavlidis S, Ng Kee Kwong F, Hoda U, Rossios C, Sun K, et al. Sputum proteomics and airway cell transcripts of current and ex-smokers with severe asthma in U-BIOPRED: an exploratory analysis. The European respiratory journal. 2018;51(5).

85. Tariq K, Schofield JPR, Nicholas BL, Burg D, Brandsma J, Bansal AT, et al. Sputum proteomic signature of gastro-oesophageal reflux in patients with severe asthma. Respiratory medicine. 2019;150:66-73.

86. Nelsestuen GL, Martinez MB, Hertz MI, Savik K, Wendt CH. Proteomic identification of human neutrophil alpha-defensins in chronic lung allograft rejection. Proteomics. 2005;5(6):1705-13.

87. Zhang Y, Wroblewski M, Hertz MI, Wendt CH, Cervenka TM, Nelsestuen GL. Analysis of chronic lung transplant rejection by MALDI-TOF profiles of bronchoalveolar lavage fluid. Proteomics. 2006;6(3):1001-10.

88. Mohammad Mohseni Majd A, Alikhani M, Mehdi Naghizadeh M, Ghazanfari T. Two dimensional proteomic analysis of serum shows immunological proteins exclusively expressed in sulfur mustard exposed patients with long term pulmonary complications. International immunopharmacology. 2020;88:106857.

89. Pajoohesh M, Naderi M, Naderi-Manesh H. Proteomic features of delayed ocular symptoms caused by exposure to sulfur mustard: As studied by protein profiling of corneal epithelium. Biochimica et biophysica acta Proteins and proteomics. 2017;1865(11 Pt A):1445-54.

90. Pashandi Z, Saraygord-Afshari N, Naderi-Manesh H, Naderi M. Comparative proteomic study reveals the molecular aspects of delayed ocular symptoms induced by sulfur mustard. International journal of proteomics. 2015;2015:659241.

91. Mehrani H, Ghanei M, Aslani J, Tabatabaei Z. Plasma proteomic profile of sulfur mustard exposed lung diseases patients using 2-dimensional gel electrophoresis. Clinical proteomics. 2011;8(1):2.

92. Mehrani H, Ghanei M, Aslani J, Golmanesh L. Bronchoalveolar lavage fluid proteomic patterns of sulfur mustard-exposed patients. Proteomics Clinical applications. 2009;3(10):1191-200.
